# A formal MIM specification and tools for the common exchange of MIM diagrams: an XML-Based format, an API, and a validation method

**DOI:** 10.1186/1471-2105-12-167

**Published:** 2011-05-17

**Authors:** Augustin Luna, Evrim I Karac, Margot Sunshine, Lucas Chang, Ruth Nussinov, Mirit I Aladjem, Kurt W Kohn

**Affiliations:** 1 Laboratory of Molecular Pharmacology, Center for Cancer Research, National Cancer Institute, NIH, Bethesda, MD 20892, USA; 2 Bioinformatics Program, Boston University, Boston, MA 02215 USA; 3 Department of Computer Engineering, Bogazici University, Bebek-Istanbul 80815, Turkey; 4 Center for Cancer Research Nanobiology Program, SAIC-Frederick, Inc., National Cancer Institute, Frederick, MD 21702, USA; 5 Department of Human Genetics and Molecular Medicine, Sackler School of Medicine, Tel Aviv University, Tel Aviv 69978, Israel

## Abstract

**Background:**

The Molecular Interaction Map (MIM) notation offers a standard set of symbols and rules on their usage for the depiction of cellular signaling network diagrams. Such diagrams are essential for disseminating biological information in a concise manner. A lack of software tools for the notation restricts wider usage of the notation. Development of software is facilitated by a more detailed specification regarding software requirements than has previously existed for the MIM notation.

**Results:**

A formal implementation of the MIM notation was developed based on a core set of previously defined glyphs. This implementation provides a detailed specification of the properties of the elements of the MIM notation. Building upon this specification, a machine-readable format is provided as a standardized mechanism for the storage and exchange of MIM diagrams. This new format is accompanied by a Java-based application programming interface to help software developers to integrate MIM support into software projects. A validation mechanism is also provided to determine whether MIM datasets are in accordance with syntax rules provided by the new specification.

**Conclusions:**

The work presented here provides key foundational components to promote software development for the MIM notation. These components will speed up the development of interoperable tools supporting the MIM notation and will aid in the translation of data stored in MIM diagrams to other standardized formats. Several projects utilizing this implementation of the notation are outlined herein. The MIM specification is available as an additional file to this publication. Source code, libraries, documentation, and examples are available at http://discover.nci.nih.gov/mim.

## Background

Diagrams have long been used to organize knowledge, and there has been an ever-growing use of such diagrams in biological sciences in the last half century. As their use increases so does the need for common methods to communicate biological knowledge accurately from author to reader in a manner similar to other disciplines that use technical drawings. Advances in molecular biology experimental techniques have resulted in an abundance of high-throughput data, placing additional emphasis on the need for the organization and visualization of biological data. Since its inception in 1999, the Molecular Interaction Map (MIM) notation has helped address this need for standardized representation of biochemical and cellular processes through a notation that shares visual characteristics with electrical circuit diagrams [1]. The notation has been featured in a variety of publications as the mechanism used to organize biological information and the basis for mathematical simulations [2-14]. The MIM notation has also garnered wide attention in the systems biology community. It has been advocated as a notation for graphical display of purely textual datasets such as those based on the BioPAX ontology [15]. The notation has also helped spur the creation of the Systems Biology Graphical Notation (SBGN) consortium, which uses the MIM notation as a basis for its SBGN Entity-Relationship (SBGN ER) language [16,17]. Potentially, the two graphical notations may converge.

As the amount of data in MIM diagrams increases, the availability of that data becomes a priority because the diagrams can serve as sources of data to be mined. The information content of MIM diagrams is often extended with annotations containing ancillary information, such as comments, external links, and citations; annotations are denoted as labels on interaction lines [1]. Annotations provide readers with additional insight into the systems they represent, which may not be captured by the MIM glyphs per se (e.g. information regarding time, location, and sequence of events). These annotations have recently been mined in the work by McIntosh and Curran who built a MIM corpus that maps MIM annotations to passages from the original research articles [18].

The present work outlines an implementation of the MIM notation and provides a new specification with a series of software tools based on this specification. Presented first is the new specification that addresses previous ambiguities in the notation, provides definitions as a foundation for translation, and establishes a set of syntax rules for the validation of MIM diagrams; the specification is provided as Additional file 1. This specification forms the basis of an XML-based format that includes elements to capture both the graphical and non-graphical elements of MIM diagrams. The next topic presented is a mechanism for validating MIM datasets according to the syntax rules found in the specification. Lastly, an application programming interface (API) is provided as a support mechanism for developers to interact with specific features of the MIM format.

## Methods and Results

### Description of the Formal MIM Specification

The MIM notation has been described previously in several publications [1,19-21]. The formal implementation presented below is based on the most widely used features in the MIM notation as presented in 2006 [21], and retains the goal of the MIM notation to present unambiguous and accurate diagrams of biological systems, while simplifying the visualization of these diagrams. The complete MIM specification is provided as Additional file 1 and online on our project homepage (http://discover.nci.nih.gov/mim).

### MIM Notation Elements

**Figure 1:**
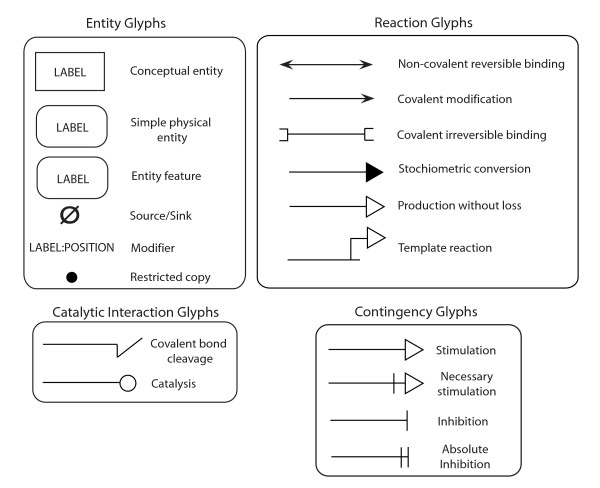
**Basic glyphs used in this implementation of the MIM notation**. The MIM notation consists essentially of glyphs representing entities and interactions; shown are the independent glyphs. A description of the usage for all the glyphs in the MIM notation is provided in Additional file 1.

MIM diagrams represent bioregulatory networks involving graphical elements broadly divided into two categories: entity and interaction glyphs. Entities may represent objects in nature with physical structures, such as biological molecules (e.g. protein, DNA, RNA, etc), or non-physical objects, such as phenotypes, behaviors, perturbations, cell cycle states, etc. Interactions are relationships between an entity and other entities or interactions. Interactions between two entities are represented in the form of binding interactions (e.g. the binding of calcium to calmodulin) or the transformation of one entity into another (e.g. the ADP and phosphate to ATP). An interaction between an entity and another interaction can be used to describe the influence the entity exerts on the interactions, such as the inhibition of binding between two proteins by a third. The basic graphical elements that represent MIM elements are referred to as glyphs and are shown in Figure 1 and full details on their usage are provided in Additional file 1.

**Figure 2:**
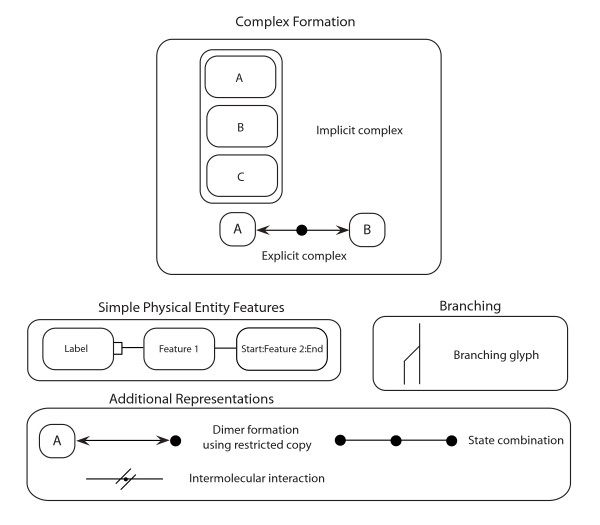
**More advanced use cases of MIM glyphs**. Examples of cases involving multiple MIM glyphs to represent a concept.

Entity glyphs are differentiated by their shapes, and the various types of interactions are represented by lines with different arrowheads or other line end-marks. For some glyphs, the semantics of the glyph are determined by the context in which the glyph is used; examples of these cases include the production without loss and stimulation glyphs. The simple physical entity and entity feature glyphs, and restricted copy entity glyphs shown in Figure 1, while explicit and implicit complex formation are shown in Figure 2. As has been the case with previous MIM specifications, the color of a glyph does not affect its semantics. The accompanying MIM specification describes the full set of MIM glyphs shown in Figures 1 and 2 along with their appropriate use.

### Entity Glyphs

The MIM notation supports various types of entities, each represented by a different glyph. The most common entity glyph is a labeled rounded-rectangle that represents a simple physical entity (SPEs) where "simple" denotes that the entity is not in a complex with other entities; this glyph is used to represent molecules such as proteins, DNA, RNA, etc. The labels of SPEs are the main identifiers used by readers in understanding MIM diagrams, and it would be helpful for the purpose of data exchange that standardized nomenclatures, such as HGNC names for genes, be used.

An SPE can also be represented in a manner that defines specific regions of molecules, such as protein domains, motifs, and sites. This representation provides a more detailed description of an SPE through the use of entity features. Entity features can be used to indicate specific regions of an SPE that carry out a particular function as illustrated in Figure 2.

SPEs are typically represented only once in a given diagram, which allows for the traceability of all the interactions of a given entity to a single location on a diagram. Modifiers are physical entities that are represented as labels without borders and generally represent small molecules (e.g. phosphate, methyl, or ubiquitin molecules) that can be depicted multiple times in a diagram. Such small molecules tend to exist frequently in pathway interactions, and it would be prohibitive to route all the connections of a small molecule to a single glyph.

**Figure 3:**
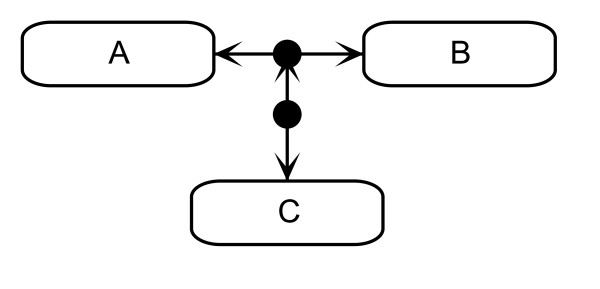
**Example of a trimer between simple physical entities A, B, and C**. The explicit complex node on the binding interaction line between entities A and B denotes the dimer A:B and the binding interaction between this complex and C denotes the trimer A:B:C.

SPEs can exist in complexes with other entities. There are two types of complexes in MIM: explicit and implicit complexes as shown in Figure 2. Explicit complexes are diagrammed as small filled circles on binding interaction lines, and are termed "explicit" because the binding partners that give rise to them are shown. Implicit complexes, however, are diagrammed as enclosures of SPEs and indicate an implied relationship between the SPEs without showing their direct interactions. Figure 3 shows a complex of SPEs A, B, and C as an explicit complex. The interaction between A and B forms the complex A:B, which is then bound to C. As represented as an explicit complex, A and B cannot be unbound if C is bound to the A:B complex; this represents a common mechanism whereby C stabilizes the A:B interaction. The notation implies that SPEs A and B must bind to each other before C can bind to them. If A and B unbind, then there would no longer be an A:B for C to bind to. Therefore, SPE C must dissociate first before A and B can dissociate. The implicit complex SPEs shown in Figure 2 does not provide readers of the MIM diagram with information on the binding order of entities unlike the explicit complex representation. This representation is useful when binding order is unknown or when those details are not relevant to the intent of the diagram.

Since SPEs tend to appear once on a diagram, it becomes necessary to have a method for diagramming homo-dimerization interactions. Such interactions make use of restricted copy entities, small black dots that act as copies of the SPEs to which they are bound. Restricted copy entities cannot be included in any other interaction; the complexes resulting from homo-dimerization interactions can participate in interactions in the same way as other explicit complexes.

There are two additional MIM entity glyphs: conceptual entity and source/sink glyphs. Conceptual entities, shown by a rectangular glyph, can be used to represent objects that do not have a clear physical structure (e.g. ionizing radiation) or whose physical structure is outside the scope of the given diagram. The source/sink symbol uses the mathematical symbol for an empty set and can be used to represent an unlimited and unspecified source for the production of an entity or an unspecified product of a degradation reaction.

### Interaction Glyphs

The notation has three categories of interactions that exist between entities: reactions (i.e. an interaction where the input and output are both entities), catalytic interactions (i.e. an abbreviation for a known set of reactions), and contingencies (i.e. an interaction in which a controller entity regulates, modifies, or otherwise influences another reaction or contingency); all the interaction glyphs are shown in Figure 1. For brevity, only the usage of a limited number of interactions is presented here. Readers are directed to the MIM specification for a description of all interaction types. The reaction types include non-covalent reversible binding, covalent modification, covalent irreversible binding, template reaction, stoichiometric conversion, and production without loss of reactants.

Non-covalent reversible binding is represented as a double-headed line with barbed arrowhead endings connecting two entities; barbed arrowheads are permissible only in accordance with the specification rules outlined in Table three of the MIM specification. The outcome of an interaction represented by a non-covalent reversible binding glyph is an explicit complex, indicated by a filled circle on the interaction line; the resulting explicit complex can then participate in additional valid interactions.

**Figure 4:**
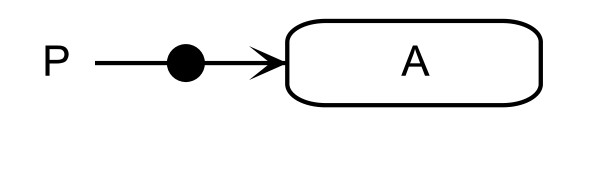
**Formation of the phosphorylated entity A**. Explicit complexes placed on the covalent modification lines represent the modified entity.

Covalent irreversible binding functions in the same manner as non-covalent reversible binding, but with different semantics, namely that those interactions must be covalent and not directly reversible. Covalent modification uses a single-headed line with a barbed ending pointing towards the modified entity with a modifier entity on the end of the line that is not barbed, as shown in Figure 4.

**Figure 5:**
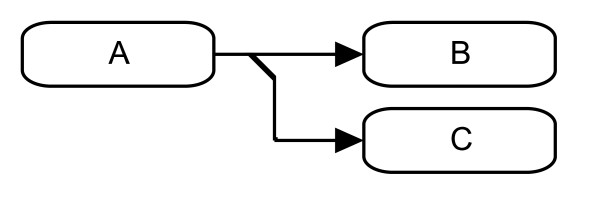
**Conversion of one entity into multiple entities**. The stoichiometric conversion of entity A to entities B and C.

Stoichiometric conversion uses a single-headed line with a triangle end that points from the reactant to the product. The stoichiometric conversion can be used to describe the production of multiple entities, as shown in Figure 5.

The MIM notation also offers glyphs to describe the stimulation and inhibition of other interactions, and these are part of the set of contingency interactions. An unfilled triangle arrowhead is used to represent stimulation, while a terminal bar is used for inhibition. To indicate that an entity is necessary for a process to occur, a bar is placed behind the unfilled triangle of the stimulation glyph.

Catalytic interactions follow a similar set of syntactic rules as contingencies, but represent interactions requiring a catalyst (e.g. the enzymatic activity of a kinase or phosphatase).

The MIM specification provides additional examples and guidance on the usage of the glyphs in the notation, especially in more complicated situations.

### Formal Rules for MIM

One of the key features of this new implementation of the MIM notation is the introduction of a strict set of syntax rules that provide constraints on valid MIM diagrams. Previous MIM publications presented both basic and elaborate examples, but did not codify the extent of the syntactic capabilities of the elements of the notation. There was no clear method to validate diagrams in terms of the manner in which elements are connected. The new syntax rules presented here will help users create and update valid MIM diagrams.

The syntax rules presented in Table three and Table four in Section 8.1 of the MIM specification treat each interaction glyph as having three possible places of connection: the start, the end, and the line itself. For symmetric interactions (e.g. non-covalent reversible binding and covalent irreversible binding), either terminus of the line may be considered the start of the interaction with respect to the syntax rules. For all other interactions types, the line terminus without an arrowhead is considered the start of the interaction. The syntax rules outline what entities may connect to the start and end of an interaction line and whether a symbol can exist on an interaction line between its termini. Additional rules in the formal MIM specification outline the usage of branching glyphs, as well as other syntactic rules.

In addition to these rules, the MIM specification outlines how glyphs should be interpreted in conjunction with other glyphs. Section 8.4 of the MIM specification specifically outlines correct interpretations of the presence of entities given the potential incompleteness of knowledge about a particular entity based on the interactions in a diagram. This is closely aligned to the ideas of the "heuristic" MIM interpretation, which recognizes that the role of "transitive" effects (the effect of a given interaction on others) is often unknown [21,22].

### Limitations of the Formal Implementation

The MIM notation formalized by the current specification is constrained to facilitate the implementation of software tools for MIM, and does not include all MIM glyphs that have been published previously. In the notation's history there have been variations in the representation of certain glyphs; this issue has been addressed by choosing a single representation for each concept in the notation. This implementation of the notation does not permit *ad hoc *glyph creation. This facilitates the validation of diagrams and allows software developers to be confident that they have implemented all known features of the implementation; other glyphs may be added in future releases of the specification.

As the notation has evolved, several shorthand notation elements have been developed to simplify common patterns in MIM diagrams. The current implementation includes only some of these shorthand notation elements from previous MIM publications. Undoubtedly, this means that certain constructs in diagrams will be more visually complex, but since these elements do not add to the semantic capacity, they have been postponed for future releases.

The specification presented here does not provide strict guidance on the glyphs of the notation that are suitable for computational simulations; this topic has been discussed previously regarding the manner in which the MIM notation may be used in conjunction with mathematical simulations [2,21]. One future development that may support such a goal is by outlining an additional validation method for glyphs appropriate in computational simulations.

Lastly, this implementation does not specify a notation to represent transport interactions. Entities in the MIM notation may represent a given entity in multiple states (e.g. a protein with some molecules in the nucleus and others in cytoplasm). With no clear way of distinguishing these states, the semantics of a transport interaction would be unclear; this is a problem common to other similar notations such as the SBGN ER Level 1 Version 1.2 notation [16]. In previous MIM publications, transport reactions have been represented by a stoichiometric conversion glyph. In many cases this representation is clear and unambiguous. That representation, however, sometimes introduces awkward ambiguities. Therefore, we did not include transport reactions in the current version of the formal MIM specifications.

### Limitations of Current Machine-Readable Representations of MIM Diagrams

Several software projects have included support for the notation and have each addressed the lack of a standardized data model for the MIM notation differently. The first associated software project for MIM diagrams integrated the diagrams with metadata in the form of E-MIMs (electronic MIMs) found at http://discover.nci.nih.gov/mim[21]. E-MIMs store the diagrams using the SVG (Scalable Vector Graphics) format to provide interactive features allowing the graphic elements to be connected to metadata. The SVG format does not retain the semantics of the elements visualized, and the metadata, currently, are reintroduced to the SVG files through a post-processing step.

Another project, the Java-based biological pathway diagram editor PathVisio, has included the glyphs of the notation for the purpose of facilitating the production of MIM diagrams [23]. The PathVisio software provides MIM-specific interaction glyphs for diagrams that are stored using the GPML (GenMAPP Pathway Markup Language) format. The MIM-specific glyphs were provided as additions to the pre-existing PathVisio glyphs that are external to the MIM notation. If users include both MIM-specific and external glyphs, a diagram will be viable in the context of the GPML format, but will lose the consistency required for exchange with other MIM-specific tools and tasks, such as validation.

Software support for MIM was recently provided by the MIMCITY database project (http://www.mimcity.org) for storing, querying, visualizing and analyzing data contained in MIM diagrams [Karac, et al., in preparation]. The project developed an accompanying data model implemented in the form of a database schema that is compatible with an SBML-based representation of the MIM notation also developed for that project. The SBML-based MIM representation addresses incompatibilities between the MIM and SBML through the use of SBML annotation containers to embed MIM information content not supported in SBML. The MIMCITY database schema and the SBML-based MIM representation, however, do not include elements to describe the visualization of MIM diagrams.

### Overview of MIM Schema

The various formats used in the aforementioned projects have limitations that highlight the need for a standard format to support future MIM software projects. The new MIM Markup Language (MIMML) meets this need and provides a standard format for the exchange of MIM diagrams among different software. The MIMML format conforms to XML Schema 1.0, and is based on the GPML schema developed for use with PathVisio [23,24]. MIM datasets are plain-text XML data streams: the datasets are characterized by matching start and end tags, and elements can contain attribute-value pairings. The schema is used to store information about the visual presentation (e.g. positioning, color, or size) and layout of the diagram as well as accompanying metadata (relationships to external databases, annotations, and citations).

**Figure 6:**
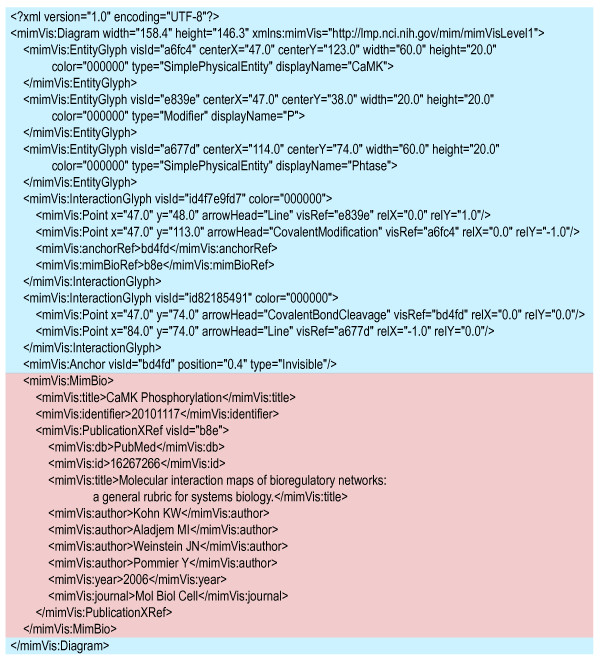
**Example MIMML dataset that describes the interaction of the cleavage of phosphate from CaMK**. Blue: Highlighted section describes graphical elements of the MIM notation. Red: Highlighted section describes metadata components of the MIM notation including diagram title and identifier and a publication cross-reference.

The MIMML format employs several XML elements of different types; the ones of most importance are outlined here. The root element of the MIMML schema is the Diagram XML element used primarily to store size information about the diagram. This element can have several types of child elements; primarily these include: EntityGlyph, InteractionGlyph, Anchor and MimBio XML elements; Figure 6 shows a small MIMML dataset that includes examples of all the MIMML elements described; other example files on the website have more comprehensive examples (http://discover.nci.nih.gov/mim). EntityGlyph is used to store information about all MIM entities. This is a departure from GPML, which stores information in implicit and explicit complexes as groups and anchors, respectively. This change allows for a uniform mechanism when validating MIM entity glyphs. InteractionGlyphs are used to store information about interactions and the elements to which they are connected. An InteractionGlyph XML element is made up of several Point elements. Point elements provide routing information and store type of arrowheads used in a given interaction as shown in Figure 6. For the purposes of validation, the MIMML schema, unlike GPML, restricts the types of arrowheads that can be used to only those that exist in the MIM notation. The first and last Points of an InteractionGlyph contain the visRef attribute to specify to which MIM element each line end is connected; shown in Figure 6. InteractionGlyphs may also include attributes pointing to Anchor XML elements. Anchors are connection points on an InteractionGlyph. These are used to connect interactions to each other, as is the case with contingencies, and also, to represent the intramolecular glyph on an interaction.

The XML elements for interactions and entities can also include references to particular metadata items stored in the MIMBio XML element that acts as the primary location for the storage of MIM metadata. The MIMML format supports two types of metadata: cross-references and annotations. Cross-references allow the mapping of external database resources to MIM elements. Annotations exist as two components: a comment and a publication cross-reference. This allows users to map particular interactions to the publications that provide evidence for the existence of the interaction, while the comments stored for specific MIM elements provide additional information about entities and interactions not captured by the notation. The structure of MIM cross-references is modeled after those in the BioPAX format [25]. The MimBio XML element also stores metadata related to the diagram, such as title and creator information; these elements are similar to those provided in GPML with the exception that they use terms from the Dublin Core set of metadata terms (http://dublincore.org/).

The MIMML schema adds metadata elements to allow controlled vocabulary to be used to describe the relationship of an external database resource and a MIM element and to allow users to specify the biological properties of entities through controlled vocabulary beyond the generic terminology used by the MIM notation (e.g. a simple physical entity can be described as a protein, DNA, RNA, etc). The values for element type were adopted from the BioPAX format to simplify the process of translating MIM diagrams to BioPAX datasets.

### An Example Diagram and MIMML Dataset

**Figure 7:**
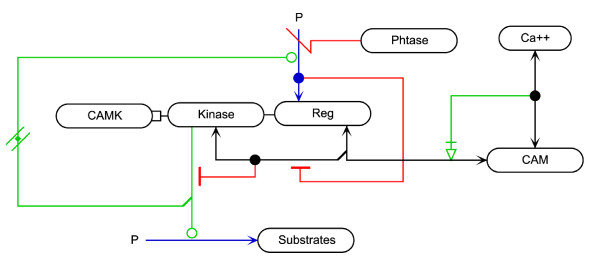
**CaMK MIM diagram based on the specification outlined in the current publication**.

In this section, we provide an example of the MIM notation and MIMML format with the use of the Ca2+/calmodulin-dependent protein kinase (CaMK) MIM diagram (shown in Figure 7) stored in the MIMML format and provided as supplemental information. The CaMK regulation MIM was originally introduced in the 2006 specification of the MIM notation as Figure twelve of that publication; a full description of the interactions is included by Kohn et al [21]. The diagram covers many of the properties of the MIM notation, thereby making it useful when describing the changes that have been made to the MIM notation. The CaMK example shows the intramolecular control of the protein kinase CaMK, and how this regulation can affect the phosphorylation of substrates (labeled here as "Substrates") by the kinase domain of CaMK. Figure 7 shows the diagram according to the formal MIM presented here. The most significant visual change is in the CaMK protein glyph. The entity glyph for the CaMK protein is linked to its two domains (the kinase and regulation domains), and the domains have been separated so they exist in two separate entity feature glyphs. The cleavage and intramolecular glyphs have undergone cosmetic changes, as well as the way that branched interactions are supported. Changes to the diagram are largely cosmetic to simplify the implementation of the notation in software editors of MIM diagrams.

### Validation of MIM Datasets

Datasets for each schema are validated at two levels: the first is with regard to the well-formedness of the dataset according the MIMML XML schema and the second are rules that are outlined in the formal MIM specification. The MIMML XML schema outlines the valid structure of a MIMML dataset, which can be used for validation purposes. The second level of rules checks proper usage of several properties of MIMML datasets: use of entity/interaction attributes, formats of labels for modifiers and entity features, use of interaction arrowheads, placement of explicit complex and intramolecular symbols, and connection of interactions to entities or other interactions. Validation of MIMML datasets against the formal MIM connection rules is done using Schematron, a rule-based validation language for finding patterns in XML trees [26]. Assertions about the presence or absence of these patterns can be used to determine that a document adheres to a given rule set. Currently, MIMML datasets are not being validated against the layout rules and recommendations found in the formal MIM specification; the focus here is to validate the syntax of MIM diagrams.

**Figure 8:**
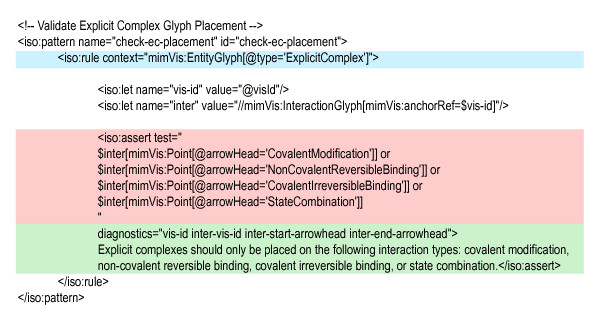
**A Schematron-formatted rule validating the placement of explicit complex glyph within a MIMML dataset**. Blue: The context to which the set of rule assertions refer; in this case, entity glyphs of type "explicit complex". Red: Assertions related to the context XML element. In this case, an explicit complex may only exist on interactions of the following types: covalent modification, non-covalent reversible binding, covalent irreversible binding, and state combination. Green: A set of diagnostic entries to be displayed, if the test assertion fails, including the ID of the explicit complex glyph (vis-id), the ID of the interaction on which the explicit complex appears (inter-vis-id), and the types of arrowheads at the start and end of the interaction glyph (inter-start-arrowhead and inter-end-arrowhead).

Figure 8 illustrates a rule for a MIMML dataset that determines whether explicit complexes in a given MIM diagram were placed on the correct types of interactions; these interaction types include: covalent modification, non-covalent reversible binding, covalent irreversible binding, and state combinations. Results from validation are returned in the Schematron Validation Report Language (SVRL), a simple report XML-based language (http://www.schematron.com/validators.html). The results provide the name of the rule fired, the elements tested, the rule tested, the location of elements failing the test using an XPath expression, and diagnostic information relevant to invalid elements.

The MIM Schematron rule set can be used wherever Extensible Stylesheet Language Transformations (XSLT) may be used with other standard XML tools. To simplify the use of the Schematron rule set, it is made available in conjunction with Java-based Schematron Ant Task (http://code.google.com/p/schematron/) along with a Java build file to show how Schematron may be used as a part of a pipeline and for the batch validation of multiple MIMML datasets.

### MIM Application Programming Interface (API) Implementation

Usage of an XML-based format to store the data of MIM diagrams allows developers to provide MIMML-related functionality using commonly available libraries capable of parsing XML data streams, but these libraries work at a low-level, at the level of XML elements and attributes. The MIM API provides a higher-level of functionality to interact directly with features of the MIMML schema.

The MIM API is a Java-based API to the elements and attributes for the manipulation and retrieval of information contained in a MIM diagram set forth by the MIMML XML schema. The interface is generated using XMLBeans (http://xmlbeans.apache.org/) a Java-to-XML binding framework used for developing Java applications built around an XML schema. The framework provides wide coverage of the features available for XML Schemas and maps XML data types to Java data types. XMLBeans generates a set of corresponding Java classes based on an input XML Schema. These generated interfaces and classes can then be used by developers to access and manipulate XML instance data using JavaBeans-style accessors (e.g. getFoo() and setFoo()), which are more friendly than usage the of the XML Document Object Model (DOM). XMLbeans provides an XML parser and validator, and it gives developers the capability of lower-level navigation of an XML document using XMLCursor. A complete description of XMLBeans is available at (http://xmlbeans.apache.org/documentation/index.html). The MIM API requires the installation of the underlying XMLBeans library (http://xmlbeans.apache.org). The XMLBeans library provides support for XPath and XQuery expressions using the Saxon XSLT and XQuery processor (http://saxon.sourceforge.net).

### Usage of the MIM API

Operations using the API are aligned to the MIM XML schema, and one Java object corresponds to each element in the MIMML document. All of the interfaces to the MIM elements inherit from the XMLObject interface, provided by XMLBeans. This interface provides basic functionality for all objects, such as the method for validation against the XML schema. Usage of the XMLBeans library provides the capability of inputting MIMML files in a variety of ways including from a file or string, but the MIM API is also capable of importing from a Java XML DOM (Document Object Model) Node object or by retrieving a MIMML data stream using a URL (Uniform Resource Locator). MIMML datasets can also be created *de novo *and existing datasets can be manipulated. The MIM API supports all the constructs of the MIMML format including the ancillary constructs, such as comments, and generic properties. Using a Java-based XSLT processing engine, such as Saxon, it is possible to additionally validate MIMML datasets against the Schematron rule set within a Java program.

### Benefits and Disadvantages of XMLBeans for the MIM API

The MIM API can be used in conjunction with APIs for other formats or libraries providing other functionality, but the major distinction between the SBML and CellML APIs and the MIM API is the usage of an XML-binding framework, which comes with both benefits and disadvantages. One benefit is that the usage of XMLBeans has simplified and sped up the development of a MIM API. This has allowed the MIM API to reach its intended audience faster, which allows developers to concentrate on developing applications that support the MIM notation and make use of the information content represented by the diagrams rather through manual generation of boilerplate code. Secondly, bugs are minimized due to the stability of the XMLBeans code base resulting from over five years of development and use. Lastly, maintainability is improved through code generation that aids in the adaption of software to future changes made to the underlying MIM schemas. One disadvantage in using XMLBeans is that it provides functionality for a single programming language, which may be a deterrent to some developers. Both SBML and CellML provide several language bindings [27,28]. As the need for the support of other languages increases, other XML-binding libraries will be used to make these language bindings available.

## Discussion and Conclusions

The Molecular Interaction Map (MIM) notation provides a way to depict bioregulatory network diagrams in a standardized manner. The notation was originally developed in 1999 and has since been further developed and updated, most notably in 2006 [1,21], where a detailed description of the glyphs and their usage was provided. The MIM notation has been developed in a fluid manner that has allowed it to depict a wide range of biological concepts including notation for polymerase, helicase, and primase activity as well as other symbols [3]. These fluid advancements in the notation enhance the range of biological networks that can be diagrammed, but can hamper the development of consistent software for the MIM notation. Here we present a well-defined and internally consistent MIM formalism and set of tools that can facilitate the development of software supporting the major parts of the MIM notation in the areas of creation, validation, and analysis of MIM diagrams. These tools should also facilitate in translation of MIMML-formatted diagrams to and from other formats, such as the BioPAX or the Systems Biology Graphical Notation Markup Language (SBGN-ML) format currently in development (http://libsbgn.sf.net/). SBGN-ML and other developments by the libSBGN group will provide developers with tools similar to the ones we present here for MIM, so that pathway editors, such as CellDesigner, Edinburgh Pathway Editor, PathVisio, and VANTED, can support SBGN in a common manner [23,29-31].

A MIM diagram editor, MIMTool, has been developed, which supports the MIMML format presented herein (http://code.google.com/p/mimtool/) [Edes, et al., in preparation]. It is limited in that it does not yet support the metadata components of the format. MIMTool is associated with the MIMCITY database for MIM diagrams, which is expected to also support the MIMML format in the future (http://www.mimcity.org) [23]. Additionally, PathVisio (http://www.pathvisio.org) is in the process of being extended to support the MIM specification and the MIMML schema; one key feature being added to PathVisio is the ability to render MIMML files (http://discover.nci.nih.gov/mim/). The example figures associated with the current publication have been produced in PathVisio [32].

The current work places a major emphasis in providing developers with basic tools to facilitate software development and enhances the level of detail for the presentation of MIM concepts. It is hoped that this new level of detail simplifies the adoption of additional MIM concepts into the SBGN notations or other notations. As one example, MIMs can represent protein domains as entity features, and this capability is important for the depiction of many critical biological signalling pathways. Interactions involving domains can therefore be represented with greater flexibility using MIMs than with SBGN ER Level 1 Version 1.2, which currently does not address domain representation [16]. For an example of how SBGN addresses interactions involving the domains represented in MIMs, the reader might compare Figure 7 for CAMK regulation in the current paper with Figure 2.1 in the SBGN ER specification. While graphical notations in biology have received strong attention in recent years, no notation has yet met all the needs of users. One of the most recent MIM publications [3] outlines the depiction of several new glyphs for polymerase, helicase, and primase activity, which helps to further the discussion on use cases still requiring a standardized depiction.

Further developments may add new components to the specification and the MIMML schema as the usage of these components of the MIM notation becomes clarified. Additionally, the combinatorial interpretation mode of MIM diagrams [22] is in the process of being algorithmically defined and will be supported in future software.

The work presented here makes advances in the usage of the MIM notation to visualize data in a way that is more "natural" to humans while retaining the qualities of being consistent and machine-readable. Participation by software developers within our group and collaborators has helped to ensure that all elements have a straightforward implementation. This implementation of the MIM notation will continue to expand to cover more of the glyphs outlined in publications on the MIM notation; each acting as a basis for the development of MIM software support.

### Availability and Ongoing Support for the MIM Specification and Software

The schemas and API are free and open source projects under the Apache License 2.0 that allows users to freely copy, distribute, and modify the projects and the underlying source code; this software may also be used in proprietary software. All project files are stored in our SVN repository and links to specific files, such as the MIMML XML Schema, are provided from the project homepage. The MIMML XML Schema is provided with documentation in the form of a webpage outlining the various XML elements and their attributes. Sample MIMML datasets are provided along with a Java Ant build file, which incorporates the Schematron Ant Task to validate the samples according to the MIM validation rules; this provides a mechanism to enhance the quality of MIM diagrams. This is a stable API for the MIMML format meant for widespread use. Documentation of the attributes and operations used in the API is provided using Javadoc (http://java.sun.com/j2se/javadoc/) on the project's SVN repository. The projects may be updated regularly to support new features, and contributions are welcome.

## Availability and requirements

• Project name: MIM Specification, API and Validation Rule Set

• Project home page: http://discover.nci.nih.gov/mim; SVN repository: https://ncisvn.nci.nih.gov/svn/mim

• Operating system(s): Platform independent. It has been tested on Mac OS X and Windows.

• Programming language: Java

• Other requirements: Java 1.5 or higher, XMLBeans 2.4.0, ISO Schematron

• License: Apache License, Version 2.0

• Any restrictions to use by non-academics: Redistribution requires compliance with the Apache License, Version 2.0.

The project homepage provides links to project resources (specification, source code, documentation, and examples) of this project. Source code for the various components is available via SVN at https://ncisvn.nci.nih.gov/svn/mim.

## Abbreviations

API: Application Programming Interface; ATP: Adenosine triphosphate; ADP: Adenosine diphosphate; BioPAX: Biological Pathway Exchange Language; CaMK: Ca2+/calmodulin-dependent protein kinase; CellML: Cell Markup Language; DOM: Document Object Model; DNA: Deoxyribonucleic acid; ER: Entity-Relationship; GenMAPP: Gene Map Annotator and Pathway Profiler; GPML: GenMAPP Pathway Markup Language; HGNC: HUGO Gene Nomenclature Committee; HUGO: Human Genome Organisation; MIM: Molecular Interaction Map; MIMML: Molecular Interaction Map Markup Language; RNA: Ribonucleic acid; SBGN: Systems Biology Graphical Notation; SBGN-ML: Systems Biology Graphical Notation Markup Language; SBML: Systems Biology Markup Language; SPE: Simple Physical Entity; SVG: Scalable Vector Graphics; SVN: Subversion; SVRL: Schematron Validation Report Language; URL: Uniform Resource Locator; VANTED: Visualization and Analysis of Networks containing Experimental Data; XML: Extensible Markup Language; XSLT: Extensible Stylesheet Language Transformations

## Authors' contributions

AL created and tested the MIM API, MIMML schema, and MIM Schematron ruleset, wrote project documentation and example MIMML datasets, edited the specification, and wrote the manuscript. MS tested the MIM API and edited the specification. LC created example MIMML datasets and edited the specification. EIK, MIA, and KWK edited the specification and manuscript. All authors have read and approved the final manuscript.

## Supplementary Material

Additional file 1 **Formal MIM Notation Specification**. Formal MIM specification documenting MIM glyphs and their usage. Click here for file
