# Supplementary material for: A formal MIM specification and tools for the common exchange of MIM diagrams: an XML-Based format, an API, and a validation method
Source: BMC Bioinformatics. 2011 May 17;12:167. doi: 10.1186/1471-2105-12-167 (PMC3118169; doi:10.1186/1471-2105-12-167)
Supplement: Additional file 1 — Formal MIM Notation Specification. Formal MIM specification documenting MIM glyphs and their usage. [file 1471-2105-12-167-S1.PDF]

Formal MIM Notation Specification

Version: Level 1, 1.0

Date: December 28, 2010

Editors

Augustin Luna[1,2], Evrim I Karac[3], Margot Sunshine[1], Lucas Chang[1], Mirit I. Aladjem[1] and Kurt W. Kohn[1]

[1]Laboratory of Molecular Pharmacology, Center for Cancer Research, National Cancer Institute, NIH, Bethesda, MD 20892, USA
[2]Bioinformatics Program, Boston University, Boston, MA 02215, USA
[3] Department of Computer Engineering, Bogazici University, Bebek-Istanbul 80815, Turkey

Contents

Editors 1

1. Introduction 6

2. Formal MIM Levels and Versions 6

3. Development of the MIM Specification Document 7

3.1 Citing this Document 7

4. Keywords, Typographical Conventions, and Controlled Vocabulary 7

4.1 Specification Keywords 7

4.2 Glyph Conventions 7

4.3 Specification Typographical Conventions 7

4.4 Controlled Vocabulary 7

4.4.1 MIM Element Controlled Vocabulary 7

4.4.2 Covalent Modification Controlled Vocabulary 8

5. MIM Glyphs 9

6. Entity Glyphs 9

6.1 Nature of Formalized MIM Entities 9

6.2 Entities 10

6.2.1 Simple Physical Entity 10

6.2.2 Entity Feature 10

6.2.3 Implicit Complex 11

6.2.4 Conceptual Entity 11

6.2.5 Modifier 12

6.2.6 Source/Sink 12

6.2.7 Explicit Complex 13

6.2.8 Restricted Copy Dot Entity 14

7. Interactions 14
7.1 General Properties of Interactions 15
7.1.1 Intermolecular Interactions 15
7.1.2 Annotations 17
7.2 Reactions 17
7.2.1 Non-Covalent Reversible Binding 17
7.2.2 Covalent Modification Binding 18
7.2.3 Covalent Irreversible Binding 19
7.2.4 Production Without Loss of Reactants 19
7.2.5 Template Reaction 20
7.2.6 Stoichiometric Conversion 21
7.3 Contingencies 21
7.3.1 Stimulation 22
7.3.2 Necessary Stimulation 22
7.3.3 Inhibition 23
7.3.4 Absolute Inhibition 23
7.4 Catalytic Interactions 24
7.4.1 Catalysis 24
7.4.2 Covalent Bond Cleavage 25
7.5 Connector Glyphs 25
7.5.1 First Feature Connector 25
7.5.2 Next Feature Connector 26
7.5.3 Simple Physical Entities with Entity Features 27
7.6 Additional Glyphs 28
7.6.1 State Combinations 28
7.6.2 Branching Glyph 29

7.6.3 Competitive Binding 30
8. MIM Language Rules: Syntax and Semantics 31
8.1 Interaction Syntax Rules 31
8.2 Additional Syntactic Rules 33
8.3 Validation of MIM Diagrams 35
8.4 Semantics in MIM 35
8.4.1 Inference Cases in MIM 37
8.4.2 Semantic Validity of MIM Diagrams 43
8.4.3 Relation to Previously Described Interpretations of MIM Diagrams 44
9. Layout Guidelines of MIM Diagrams 44
9.1 Layout Requirements 44
9.1.1 Entity-entity overlap 45
9.1.2 Arrowhead overlap 45
9.1.3 Interaction-interaction overlap 45
9.1.4 Color usage 45
9.2 Layout Recommendations 45
9.2.1 Entity labels 45
9.2.2 Interaction annotation labels 45
9.2.3 Label orientation 46
9.2.4 Single line labels 46
9.2.5 Interaction line routing 46
9.2.6 Node duplication 46
9.2.7 Ordering of entity features 46
9.2.8 Labeling of covalent modifications 46
9.2.9 Label overlap 47
9.2.10 Minimizing line length 47

10. Known Issues 47
10.1 Interpretation Modes 47
10.2 Glyph Set 47
10.2.1 Expanded Concepts 47
10.2.2 Shorthand Glyphs 48
11. Quick Reference Sheet 48
12. Revision History 50
13. Acknowledgements 50
13.1 Main Contributors 50
13.2 Acknowledgements 50
14. References 50

1. Introduction

The Molecular Interaction Map (MIM) notation seeks to provide a standardized method to draw diagrams for bioregulatory networks with features similar to technical diagrams in other fields. The document draws on work initiated by Kurt Kohn in the development of the MIM notation that was first published in 1999 (Kohn 1999). Work on and related to the MIM notation has been continued in subsequent publications (Kohn 2001; Aladjem, Pasa et al. 2004; Kohn, Aladjem et al. 2006; Kohn, Aladjem et al. 2009). Here we extend the MIM notation by providing detailed semantics and syntactic rules for the glyphs of the MIM notation. This specification provides support for the majority of features included in the last fully described MIM specification (Kohn, Aladjem et al. 2006). This extension is a necessary addition to allow for the validation of the MIM diagrams and to facilitate software development and translation of the notation to other languages. Figure 1 shows the regulation of CaMK as MIM diagram; this is a representative diagram in that it utilizes the many of the glyphs in the notation.

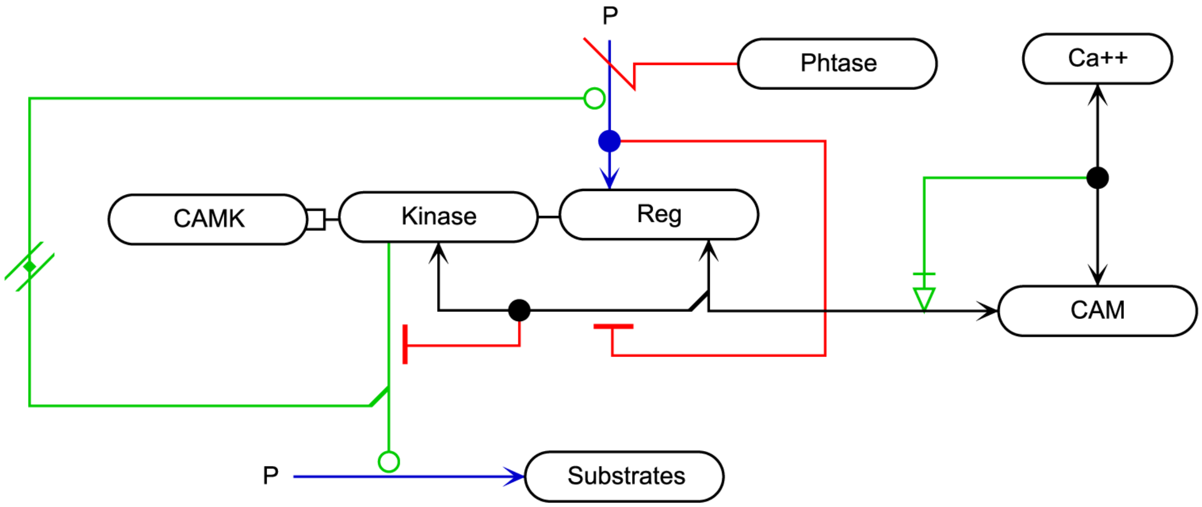

Figure 1: CaMK regulation as MIM diagram.

2. Formal MIM Levels and Versions

The formal MIM specification continues to be developed and expanded as the semantic and syntactic properties of previously described MIM glyphs are clarified. The formal MIM specification follows a pattern of versioning similar to other related projects, including BioPAX, SBGN, and SBML, through the use of levels and versions. Levels constitute usable sets of glyphs with prescribed properties. Each level may possess incremental versions that refine the semantic or syntactic properties of the glyphs in a given level. New glyphs may also be added within the incremental versions, but the addition of broad categories of functionality to the notation are set aside for future levels. Glyphs whose semantic and syntactic properties are still being developed will be postponed for inclusion in future levels (see Section 10 for known issues with the notation).

3. Development of the MIM Specification Document

Materials pertaining to the MIM notation are located at http://discover.nci.nih.gov/mim, and updates to these documents will be posted on this site. The latest version of this document can be found here: http://discover.nci.nih.gov/mim Comments, feedback, and suggestions about this document may be sent to webadmin@discover.nci.nih.gov.

3.1 Citing this Document

This document may be cited in the following manner:

Luna et al. (2010) Formal MIM Notation Specification Level 1. http://discover.nci.nih.gov/mim

4. Keywords, Typographical Conventions, and Controlled Vocabulary

4.1 Specification Keywords

The keywords "MUST", "MUST NOT", "REQUIRED", "SHALL", "SHALL NOT", "SHOULD", "SHOULD NOT", "RECOMMENDED", "MAY", and "OPTIONAL" shall be interpreted as described in RFC 2119 (Bradner 1997).

4.2 Glyph Conventions

One convention relates to interaction glyphs which are lines connecting two other MIM glyphs and provides information about how the two glyphs interact. Each interaction glyph has a start and an end; the convention used for the purposes of the following rules is that the "start" is the left-side end of the glyph and the "end" is the right-side of the glyph, as presented in this document. For symmetric interaction glyphs, either side of an interaction glyph may be considered the "start" or "end." "On" means anywhere within the interaction line except for at its ends.

4.3 Specification Typographical Conventions

Some graphics in the specification use an italicized serif font to highlight specific components on the examples; these labels carry no meaning and are ancillary to the notational elements outlined in this specification.

Binding between entities may be indicated using a colon. For example, a complex with A, B, C may be denoted as "A:B:C". A modification of an entity may be specified using a period. For example, an entity A which has been phosphorylated (see Section 4.4.2) may be denoted as "A.P"; the modification will appear to the right of the period. Multiple modifications may be appended in this manner. For instance, A.P.Me, indicates that entity A is both phosphorylated and methylated. This is a limited typographical notation that may not scale to large examples.

4.4 Controlled Vocabulary

Where possible, Systems Biology Ontology (SBO) and Biological Pathway Exchange (BioPAX) terms are used. (Le Novere 2006; Le Novère 2006; Demir, Cary et al. 2010).

4.4.1 MIM Element Controlled Vocabulary

Descriptions of MIM elements use the following vocabulary definitions to describe the role of the MIM elements in interactions:

- CONTROLLED: Referring to an interaction or conceptual entity that is the target of a contingency interaction.
- CONTROLLER: Referring to a source entity of a contingency or catalytic interaction.
- PRODUCT: Referring to an entity that is formed as a result of a reaction.
- REACTANT: Referring to an entity that is used as a substrate in a reaction.

These terms are the same as those used in BioPAX ontology. These terms provide information about the role of a MIM element in an interaction. As parts of language these terms may be used as adjectives (e.g. a product entity of a stoichiometric reaction).

4.4.2 Covalent Modification Controlled Vocabulary

To improve readability between diagrams, the following labels should be used for the given modification types; these are the same as used in the Systems Biology Graphical Notation Entity-Relationship language (Le Novère 2009).

Table 1: Common values for covalent modification labels

Modification Type	Label	SBO Term
**Acetylation**	Ac	SBO:0000215
**Glycosylation**	G	SBO:0000217
**Hydroxylation**	OH	SBO:0000233
**Methylation**	Me	SBO:0000214
**Myristoylation**	My	SBO:0000219
**Palmytoylation**	Pa	SBO:0000218
**Phosphorylation**	P	SBO:0000216
**Prenylation**	Pr	SBO:0000221
**Protonation**	H	SBO:0000212
**Sulfation**	S	SBO:0000220
**Ubiquitination**	Ub	SBO:0000224

5. MIM Glyphs

MIM elements are represented by glyphs split into two main categories: entity glyphs and interaction glyphs. Section 6 describes entity glyphs and Section 7 describes interaction glyphs; further information about interactions may be specified using general glyphs outlined in Section 7.1. The interaction glyphs include three main categories: reactions, catalytic interactions, and contingencies; there are several additional interaction glyphs not covered within these categories (see Section 7.6). These additional glyphs serve several purposes: 1) to describe the relationship of multiple entities in terms of time or space, 2) to describe the organizational relationships of multiple entities, and 3) to describe common patterns found in diagrams and reduce thereby visual complexity of MIM diagrams. Section 8 describes the rules for combining MIM glyphs into valid statements. Section 9 describes requirements and recommendations for the visual layout of MIM diagrams. A quick reference sheet with glyphs of the MIM notation is provided in Section 11.

Table 2: Role of formal MIM notation glyphs

Type	Role	Examples
**Entity**	Something that exists with a physical structure or as a concept	A protein or a pathway
**Reaction**	An interaction with a defined chemical mechanism	Binding between two proteins
**Catalytic Interaction**	An interaction involving an enzyme	Catalysis
**Contingency**	An interaction with a poorly defined chemical mechanism	Stimulation or inhibition
**Additional**	Other relationships between MIM elements.	The first or next feature connectors

6. Entity Glyphs

6.1 Nature of Formalized MIM Entities

Entities in MIM are abstractions of things found in nature. MIM entities represent pools of entities of the same type. Distinctions are made between entities with a physical structure (simple physical entities, explicit complexes, implicit complexes, restricted copies, source/sink, and modifiers) and those without a physical structure (conceptual entities). Entity glyphs that represent objects with physical structure can be used to represent any type of biological molecule (e.g. protein, DNA, RNA, etc). Entities are placed either at the start, end, or on the lines of appropriate interaction lines (see Section 8) as the sources, targets, or outcomes of interaction behaviors; the start and end glyphs are the interactors of the interaction.

6.2 Entities

6.2.1 Simple Physical Entity

Definition:
A pool of molecular species with a physical structure.

Glyph:
Simple physical entities are represented as rounded corner rectangles.

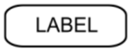

Figure 2: Simple physical entity

Label:
Labels of simple physical entities are presented as strings of characters.

Note(s):

- Molecules that act as modifiers of many simple physical entities should be represented as Modifiers (see Section 6.2.5).

- Specific regions within simple physical entities should be represented using Entity Features (see Section 6.2.2).

6.2.2 Entity Feature

Definition:
A linear part of a simple physical entity that can participate in interactions (e.g. domain, motif, or site).

Glyph:
Entity features are represented as rounded corner rectangles and are distinguished from simple physical entity glyphs based on their interactions with other entities. Entity features should be connected by a first feature or next feature glyph according to the rules in Section 8.

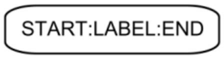

Figure 3: Entity feature

Label:
Entity features should include a horizontal label as the main label of the entity represented as a string of characters, and may include two additional labels: (1) an optional label on the left of the entity indicating the start location of the entity feature and (2) an optional label on the right of the entity indicating the end location of the entity feature. These optional labels should be integers and are demarcated using colons to separate them from the main label. An entity feature may only have one set of start and end locations. If the two labels are the same, then a single

position is represented. Since features may overlap, their position numbers may have overlapping values.

Note(s):

- Overlap of start and end position labels can be used to indicate features within other entity features.
- Entity features should not exist in the absence of a simple physical entity of which they are a part of.

6.2.3 Implicit Complex

Definition:
A complex physical entity where interactions between the contained simple physical entities is implied, but not explicitly represented.

Glyph:
Implicit complexes are rounded corner containers around a set of simple physical entities.

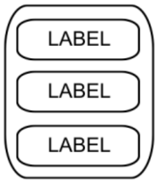

Figure 4: Implicit complex

Label:
Possesses no label of its own

Note(s):
Direct interactions between simple physical entities in an implicit complex are not known unless explicitly represented.

6.2.4 Conceptual Entity

Definition:
A non-physical entity, such as phenotypes, actions, behaviors, perturbations, cell cycle states.

Glyph:
A rectangular box enclosing a label

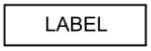

Figure 5: Conceptual Entity

Label:
Labels of simple physical entities are presented as strings of characters.

Note(s):

- Conceptual entities may be used to represent any concept. For instance, a protein may be represented as a conceptual entity. In such a case, the protein would be restricted to the semantics of a conceptual entity and restricted to participating in interactions defined for conceptual entities.

6.2.5 Modifier

Definition:
A physical entity that covalently modifies a simple physical entity or an entity feature.

Glyph:
Modifiers are unenclosed labels.

$\text{LABEL}^{\text{POSITION}}$

Figure 6: Modifier with superscripted position label

LABEL:POSITION

Figure 7: Modifier with demarcated position label

Label:
To improve readability between diagrams, the labels from Section 4.4.2 (e.g. P, Me, OH) should be used for the given modification types; these are the same as used in the Systems Biology Graphical Notation (Moodie 2009). The optional position label indicates the location on a simple physical entity where the modification is bound; recommendations regarding the position label are in Section 9.2.8. This label may either be superscripted in proximity to the main label, as shown in Figure 6, or may be demarcated using a colon, as shown in Figure 7.

Note(s):

- Modifiers must interact with simple physical entities through a covalent modification interaction. Modifiers typically represent small molecules; any small molecule that is not involved in a covalent modification should be represented as a simple physical entity.

6.2.6 Source/Sink

Definition:
A pool of molecular species that acts as either a source or a sink for the production or degradation of another entity. The nature of this pool of entities is outside the scope of a given diagram.

Glyph:
The entity is represented as the empty set symbol commonly used in mathematical notation.

$\varnothing$

Figure 8: Source/Sink

Label:
Possesses no label

Note(s):
- Source/sink glyphs should only participate as an interactor in a single interaction. For guidelines regarding node duplication see Section 9.2.6.

6.2.7 Explicit Complex

Definition:
A complex physical entity created as a result of a binding interaction between two entities (e.g. a protein complex).

Glyph:
Filled circle placed on an interaction line.

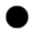

Figure 9: Explicit complex

Label:
Possesses no label

Example(s):
Explicit complexes can be used to describe the formation of heterodimers.

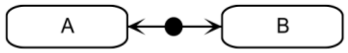

Figure 10: Example of complex formation between simple physical entities A and B

Note(s):
- Use of explicit complexes allows the MIM notation to be extensible. For example, a node that represents a dimer may be connected to another entity to represent a trimer. In Figure 11, The explicit complex on the binding interaction line between entities A and B denotes the dimer A:B and the binding interaction between this and C denotes the trimer A:B:C

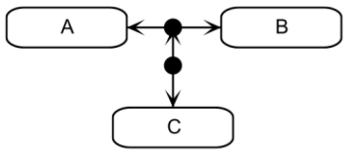

Figure 11: Example of a trimer between simple physical entities A, B, and C.

- Explicit complexes placed on the covalent modification lines represent the modified molecules as shown in Figure 12.

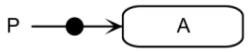

Figure 12: Formation of the phosphorylated entity A

- Multiple explicit complexes may be placed on a single binding interaction.
- Multiple explicit complexes on an interaction imply the same instance of an entity.
- In the trimer A:B:C, shown in Figure 11, no information is given as to whether entity C binds to entity A or B.

6.2.8 Restricted Copy Dot Entity

Definition:
A copy of the simple physical entity represented at the other end of a binding interaction.

Glyph:
A filled circle that is connected to one end of a reversible non-covalent binding interaction glyph

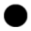

Figure 13: Restricted Copy

Label:
Possesses no label

Example(s):
Restricted copy dot entities should be used to describe the formation of homodimers; the resulting product of the binding interaction in Figure 14 is the complex A:A.

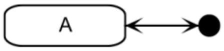

Figure 14: Homodimer formation

Note(s):

- Simple physical entities typically exist once per diagram; see Section 9.2.6. The MIM notation provides a means of representing homodimer using a restricted copy dot entity. A non-covalent reversible binding between a simple physical entity and a restricted copy dot entity results in a homodimer.

7. Interactions

An interaction is a relationship between two entities or an entity and another interaction that can occur if the potentially interacting entities are present at the same time and in the same place. There are three main categories of interactions: reactions, catalytic interactions, and contingencies. Interactions are distinguished by different arrowheads placed at the ends of interaction lines pointing to the interactor glyphs. Binding interactions (with the exception of

covalent modification) are symmetric interactions that possess identical arrowheads at each end. All other interaction types are asymmetric. Multiple interactions may be combined using the branching glyph (Section 7.6.2) with restrictions outlined in Section 8.

Note(s):

- The line segments associated with interaction glyphs that cross do not affect the meaning of the crossing interactions; it is as if they do not touch; see 9.2.5 for guidelines regarding interaction routing.

- Interactions in MIM diagrams occur *in cis*, meaning that a single instance of an entity is involved, unless the intermolecular glyph is used. Binding interactions must possess distinct instances of an entity or features of an entity as the interactors. One way that this may be done is through the intermolecular glyph discussed in section 7.1.1 or through the use of the restricted copy entity 6.2.8. The explicit complexes shown as incorrect in the Figure 15 represent the same instance of an entity and may not bind to itself.

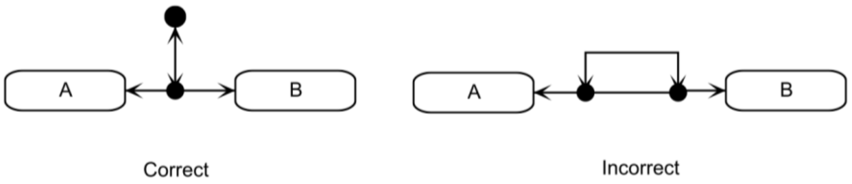

Figure 15: Distinct and indistinct entities.

7.1 General Properties of Interactions

7.1.1 Intermolecular Interactions

Definition:
Indication that an interaction occurs between different instances of the same entity

Glyph:
Two slashes leaning in the same direction placed on an interaction line with a point between the two slashes. The orientation of the intermolecular glyph on the interaction line has no bearing on its semantics.

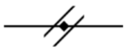

Figure 16: Intermolecular glyph

Label:
Possesses no label

Example(s):
Figure 17 shows the binding of two entity features on different instances of entity A; entity feature B of entity A binds entity feature C of a different instance of entity A.

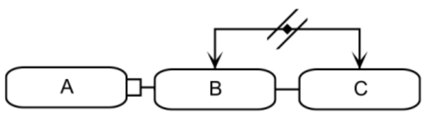

Figure 17: The intermolecular non-covalent reversible binding of features B and C of entity A.

Figure 18 shows the catalysis of the phosphorylation of entity A by a second instance of entity A.

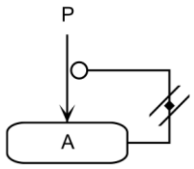

Figure 18: Intermolecular catalysis example.

As stated in Section 7, interactions in MIM exist *in cis* unless the intermolecular glyph is used. Figure 19 illustrates the disambiguation between *in cis* and *in trans* interactions. The interaction labeled "x" of Figure 19 shows that entity A in complex with entities B and C catalyzes the stoichiometric conversion of the A:B:C complex. The interaction labeled "y" of Figure 19 shows that an entity A separate from a complex A:B:C performs the catalysis.

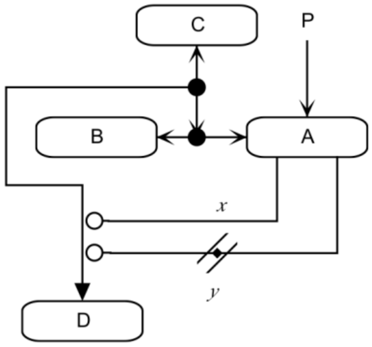

Figure 19: *in cis* and *in trans* comparison for interactions.

Note(s):

- Use of the intermolecular glyph on catalytic interactions or contingencies on a reaction refers to the interactors of the targeted reaction and the origin of the interaction carrying the intermolecular glyph.
- Use of the intermolecular glyph on catalytic interactions or contingencies on another catalytic interaction or contingency refers to the origins of both the targeted reaction and the interaction carrying the intermolecular glyph.

7.1.2 Annotations

Definition:
Interaction glyphs may be labeled; these labels specify a particular interaction and indicate that there are comments and/or citation information associated with this interaction.

Label:
An annotation is an unbounded label placed close to an interaction line.

Example(s):

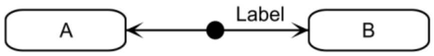

Figure 20: An interaction with the annotation label "Label"

Note(s):
- These interaction labels may be chosen by the user, but are not used as unique identifiers for interactions.

7.2 Reactions

Definition:
An interaction with a defined chemical mechanism or mechanisms where both the source and target of the interaction are entities. The outcome of binding interactions (i.e. non-covalent reversible, covalent irreversible, and covalent modification) is represented by using an explicit complex; small filled circles placed on the binding interaction line.

Note(s):
- The entities at the start and end of a binding reaction are the reactants of the interaction and the explicit complex Section 6.2.7 on the interaction line is the product of interaction.

- The entity at the start of a non-binding reaction represents the reactant while the entity at the end of reaction represents the product of the reaction.

7.2.1 Non-Covalent Reversible Binding

Definition:
A binding interaction between two physical entities resulting in the formation of an explicit complex that is reversible without an external factor (e.g. protein complexes).

Start:
Entities according to the rules of Table 3

End:
Entities according to the rules of Table 3

Glyph:
An interaction line with barbed arrows at each end.

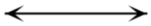

Figure 21: Non-covalent reversible binding

Example(s):
The formation of a complex using the non-covalent reversible binding interaction is shown in Figure 22.

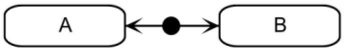

Figure 22: Example of complex formation between simple physical entities A and B

Note(s):
- A non-covalent reversible binding interaction line between two entities implies the existence of an explicit complex regardless of whether an explicit complex symbol is shown on the binding line.

7.2.2 Covalent Modification Binding

Definition:
An asymmetric covalent non-reversible binding reaction (e.g. phosphorylation and acetylation) where one of the substrates must be a modifier physical entity.

Start:
Entities according to the rules of Table 3

End:
Entities according to the rules of Table 3

Glyph:
An interaction line with a barbed arrowhead at one end and no arrowhead at the other end.

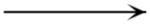

Figure 23: Covalent modification

Example(s):
- Explicit complexes placed on the covalent modification lines represent the modified molecules as shown in Figure 24.

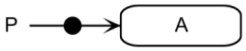

Figure 24: Formation of the phosphorylated entity A

Note(s):
Modifications may not be chained on to other modifications; Figure 25 shows the representation of an entity bound to multiple ubiquitin entities.

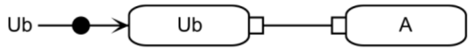

Figure 25: Formation of an entity modified by multiple ubiquitin entities.

7.2.3 Covalent Irreversible Binding

Definition:
A symmetric covalent and irreversible binding interaction between physical entities.

Start:
Entities according to the rules of Table 3

End:
Entities according to the rules of Table 3

Glyph:
An interaction line with open square arrowheads at each end

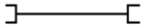

Figure 26: Covalent irreversible binding

Example(s):

- Entities A and B are bound the covalent irreversible binding interaction.

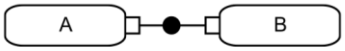

Figure 27: Covalent irreversible binding between entities A and B

Note(s):

7.2.4 Production Without Loss of Reactants

Definition:
An interaction in which a reactant entity is the basis for the formation of another entity without the loss of reactant mass.

Start:
Entities according to the rules of Table 3

End:
Entities according to the rules of Table 3

Glyph:
An interaction line with an unfilled triangle arrowhead at one end and no arrowhead at the other end

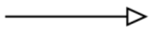

Figure 28: Production without loss of reactants

Example(s):
One use case involving this glyph is in diagramming the production of protein from a DNA template without regard for intermediate steps.

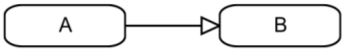

Figure 29: The production of entity B without loss in mass of entity A

Note(s):

- Transcription and translation events should be represented using the template reaction glyph when it is desired to retain such semantics.
- This glyph may also be used to describe poorly understood situations or where the details are purposely omitted in which the action of one protein increases the amount of another protein but does not reduce the quantity of the originating protein.

7.2.5 Template Reaction

Definition:
A type of production without loss that involves a template (e.g. the polymerization of a nucleic acid macromolecule from a nucleic acid macromolecule template).

Start:
Entities according to the rules of Table 3

End:
Entities according to the rules of Table 3

Glyph:
A stepped interaction line with an open triangle at one end of the line and no arrowhead at the other end. The originating line should overshoot the step with a small foot; this allows the transcription glyph to be distinguished from stimulation and production without loss glyphs.

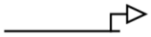

Figure 30: Template reaction

Example(s):

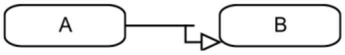

Figure 31: Transcription/translation of entity A to form entity B

Note(s):

- The template reaction glyph should be used to describe transcription or translation, but the glyph itself is insufficient to distinguish between the two biological processes.

7.2.6 Stoichiometric Conversion

Definition:
An interaction in which one entity is transformed into one or more other entities (e.g. the conversion of ATP to cyclical-AMP).

Start:
Entities according to the rules of Table 3

End:
Entities according to the rules of Table 3

Glyph:
An interaction line with a filled triangle arrowhead at one end and no arrowhead at the other end

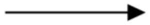

Figure 32: Stoichiometric conversion

Example(s):
The conversion of one entity into another entity:

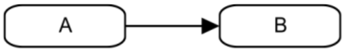

Figure 33: The stoichiometric conversion of entity A to entity B

The conversion of one entity into multiple entities:

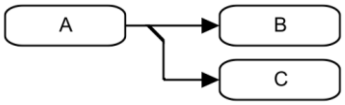

Figure 34: The stoichiometric conversion of entity A to entities B and C

Note(s):
- There is no current support to represent stoichiometric coefficients and information should not be assumed about stoichiometric coefficients.

7.3 Contingencies

Definition:

An interaction in which a controller entity regulates, modifies, or otherwise influences another reaction, catalytic interaction, contingency, or conceptual entity. The supported contingency types include: stimulation, necessary stimulation, inhibition, absolute inhibition.

7.3.1 Stimulation

Definition:
Enhancement of the velocity or extent of a reaction or contingency by the controller entity.

Start:
Entities according to the rules of Table 3

End:
Interactions according to the rules of Table 4

Glyph:
An interaction line with an unfilled triangle arrowhead at one end and no arrowhead at the other end

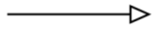

Figure 35: Stimulation

Example(s):

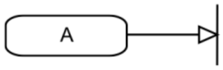

Figure 36: Entity A stimulates an interaction.

7.3.2 Necessary Stimulation

Definition:
An interaction where the controller entity is necessary for the controlled interaction to proceed.

Start:
Entities according to the rules of Table 3

End:
Interactions according to the rules of Table 4

Glyph:
An interaction line with an unfilled triangle arrowhead at one end with a bar is added behind the triangle and no arrowhead at the other end.

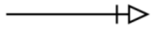

Figure 37: Necessary stimulation

Example(s):

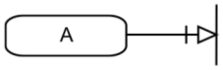

Figure 38: Entity A must necessarily stimulate the interaction for it to proceed.

Note(s):

- See Section 8.4 for details on regarding semantics.

7.3.3 Inhibition

Definition:
An interaction where a controller entity causes a decrease in the velocity or extent of controlled reaction or contingency; the probability of the controlled interaction occurring remains greater than 0.

Start:
Entities according to the rules of Table 3

End:
Interactions according to the rules of Table 4

Glyph:
An interaction line with a terminal bar at one end and no arrowhead at the other end

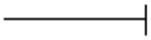

Figure 39: Inhibition

Example(s):

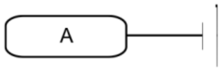

Figure 40: Entity A inhibits an interaction

7.3.4 Absolute Inhibition

Definition:
An interaction where a controller entity causes a decrease in the velocity or extent of controlled reaction or contingency; the probability of the controlled interaction is reduced to a negligible value. The target interaction does not occur if the source entity is present.

Start:
Entities according to the rules of Table 3

End:

Interactions according to the rules of Table 4

Glyph:
Represented by a line with two terminal bars pointing towards the inhibited interaction.

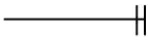

Figure 41: Absolute Inhibition

Example(s):

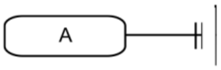

Figure 42: Entity A absolutely inhibits an interaction

Note(s):

- The semantic difference between inhibition and absolute inhibition is as follows: Inhibition means that the inhibitor reduces the magnitude or strength of the target interaction. Absolute inhibition means that the inhibitor completely prevents the target interaction from occurring.

- See Section 8.4 for details on regarding semantics.

7.4 Catalytic Interactions

Definition:
A catalytic interaction is a chemical change that is enhanced by an agent that reduces the kinetic energy barrier between reactant and product. (e.g. the enzymatic activity of a kinase or phosphatase).

7.4.1 Catalysis

Definition:
An interaction where the controller entity increases the rate of the controlled reaction.

Start:
Entities according to the rules of Table 3

End:
Interactions according to the rules of Table 4

Glyph:
Represented by an open circle at the end of the interaction line.

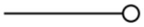

Figure 43: Catalysis

Example(s):

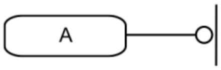

Figure 44: Entity A catalyzes an interaction

7.4.2 Covalent Bond Cleavage

Definition:
The scission of a covalent bond or the separation of connections between entity features.

Start:
Entities according to the rules of Table 3

End:
Interactions according to the rules of Table 4

Glyph:
The glyph uses two line segments; the first line segment is orthogonal to the originating line. At the end of the first line segment, a second line segment makes a 45° angle with the first segment and points towards the originating line.

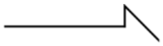

Figure 45: Covalent bond cleavage

Example(s):

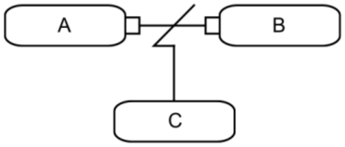

Figure 46: Entity C cleaves the covalent bond between entities A and B

Note(s):
- Cleavage of one bond might still allow a molecule to be held together by another bond. For example, a circular DNA molecule that is linearized when one phosphodiester bond is cleaved.

7.5 Connector Glyphs
Connectors are interaction glyphs that indicate organizational relationships between entities.

7.5.1 First Feature Connector

Definition:

An organizational relationship between a simple physical entity and the first feature in a chain of entity features (e.g. a series of protein domains)

Start:
Entities according to the rules of Table 3

End:
Entities according to the rules of Table 3

Glyph:
Represented by have a squared C shape pointing away from the interaction line.

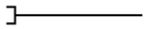

Figure 47: First feature connector

Example(s):

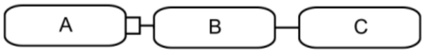

Figure 48: Simple physical entity with features. The first feature connector is represented between entity A and entity feature B.

Note(s):

- A simple physical entity can only have one first feature.
- If simple physical entity (Section 6.2.1), SPE, and entity feature (Section 6.2.2), EF are joined by a first feature glyph, then EF is the first listed region of functionality for SPE; other features might exist prior to EF that are not represented in the chain of SPE features.

7.5.2 Next Feature Connector

Definition:
An interaction that indicates the next feature in a chain of entity features (e.g. a series of protein domains).

Start:
Entities according to the rules of Table 3

End:
Entities according to the rules of Table 3

Glyph:
An arrowhead-less glyph connecting two entity features

Figure 49: Next feature connector

Example(s):

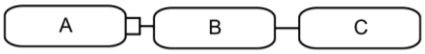

Figure 50: Simple physical entity with features. The first feature connector is represented between entity feature B and entity feature C.

Note(s):

- An entity feature can be connected to at most two other entity features or to an entity feature and a simple physical entity with a first feature connector.
- Entity features should not exist by themselves without being linked to a simple physical entity.
- If entity features (Section 6.2.2), EF1 and EF2, are joined by a next feature glyph for simple physical entity (Section 6.2.1), SPE, then EF2 is the next listed region of functionality for SPE; other unlisted features might exist prior to EF2 in the chain of SP features.

7.5.3 Simple Physical Entities with Entity Features

Simple physical entities may possess regions that participate in interactions; an example entity with features shown in Figure 49. The first entity feature connects to the simple physical entity using the first feature glyph. Subsequent features should be connected together using the next feature glyph.

Example(s):

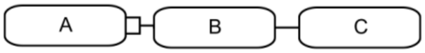

Figure 51: Simple physical entity with features.

In cases where an interaction occurs on a specific feature, it should be drawn on the feature as shown in Figure 52 for the bond formed between entity E and feature B of entity A. If the location of the interaction is unknown, the interaction line should be drawn to the originating simple physical entity, as in Figure 52 for the bond between entities A and D.

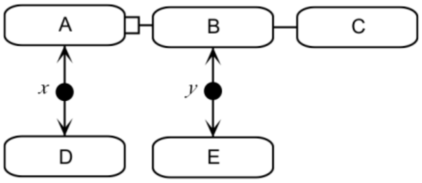

Figure 52: Binding to an entity with features. Explicit complex "x" is a complex between entity A and D and the location of the bond between A and D is not known. Explicit complex "y" is a complex between entity A and E with the bond on feature B.

Note(s):

- See Section 9.2.7 for recommendations on the order that features should be chained.

7.6 Additional Glyphs

This section contains glyphs of the MIM notation that do not fall under any other category of glyphs.

7.6.1 State Combinations

Definition:

An interaction used to represent the combination of the states of a single simple physical entity defined at the two ends of the interaction existing at the same time.

Start:

Entities according to the rules of Table 3

End:

Entities according to the rules of Table 3

Glyph:

An arrowhead-less glyph connecting two explicit complex dot entities. State combinations combine the states of two explicit complexes sharing one common simple physical entity.

Figure 53: State combination

Example(s):

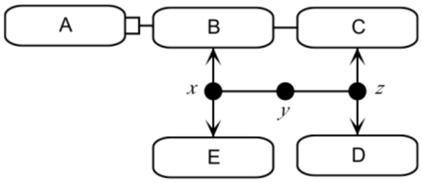

Figure 54: Combination of states with entities. Explicit complex "y" represents a pool of entities where entity E is bound to feature B and entity E is bound to feature D; features B and C are features of entity A.

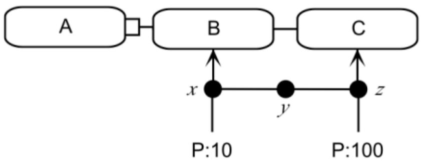

Figure 55: Combination of states with modifications. Explicit complex "y" represents a pool of entities where entity A has two simultaneous phosphorylation modifications: one at position 10 and the other at position 100 of entity A.

Note(s):

- Syntactical confusion is avoided between state combination and next feature because a state combination can only exist between two explicit complexes and the next feature glyph can only exist between two entity features.

7.6.2 Branching Glyph

Definition:
A connection point for two interaction lines

Glyph:
One interaction line may merge with (or branch from) another interaction line using a 45° line.

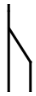

Figure 56: Branching glyph

Example(s):
Branching connectors provide a mechanism to simplify the visualization of diagrams. The semantics of the branching connector depends on the arrowheads appearing at the end of the interaction and all its branches.

Note(s):

- A contingency (Section 7.3) or catalytic interaction (Section 7.4) affects the interaction it targets and any of its child interactions. In Figure 57, stimulation "a" targets "x" thereby affecting interactions "y" and "z". Stimulation "b" targets "y" and affects only it; likewise, stimulation "c" targets "z" and affects only it.

- Each separate branch of a branching set of contingencies or catalytic interactions should be considered a separate interaction. In Figure 57, interactions "y" and "z" should be considered separate interactions.

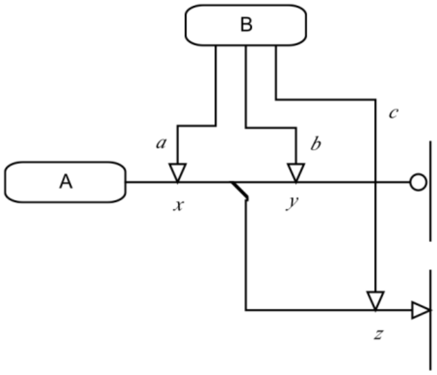

Figure 57: Interactions labeled "a", "b", and "c" target, respectively, the single interaction labeled as "x", "y", and "z".

- A branched stoichiometric conversion is understood to represent a single interaction. A contingency (Section 7.3) or catalytic interaction (Section 7.4) targeting a stoichiometric conversion affects the entire interaction no matter which branch it targets. In Figure 58, each stimulation interaction "a", "b", and "c" targets the single stoichiometric conversion interaction regardless of where it targets the interaction at points "x", "y", or "z".

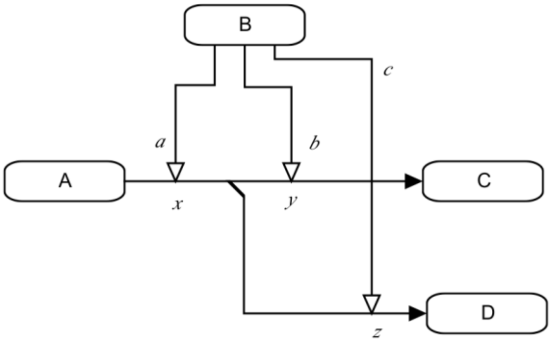

Figure 58: Interactions labeled "a", "b", and "c" target the entire interaction labeled as "x", "y", and "z".

7.6.3 Competitive Binding

Definition:

A representation depicting that the binding of one entity to another prevents the binding of the first entity to a third entity. The competitive binding representation shown in Figure 60 is a shorthand representation for the interactions in Figure 59.

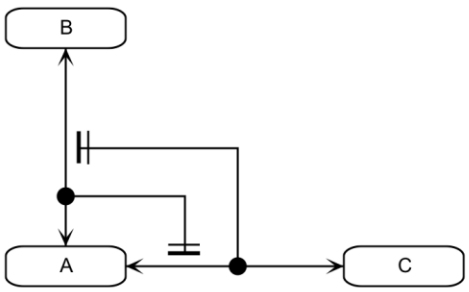

Figure 59: A set of mutual inhibition interactions that can be represented in a shorthand manner using the representation in Figure 60.

Example(s):
This representation possesses a set of combined interaction lines; each line represents a different explicit complex as shown in Figure 60.

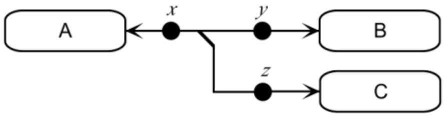

Figure 60: Competitive Binding. Nodes "y" and "z" represent the entity complexes A:B and A:C, respectively. Node "x" represents a pool of explicit complexes containing both A:B and A:C.

Note(s):

- Competitive binding should only utilize simple physical entities, entity features, or explicit complexes and must not use implicit complexes. This rule removes semantic ambiguity in the competitive binding with regards to entities within the implicit complex and their role in the competitive binding.

8. MIM Language Rules: Syntax and Semantics

This section describes how formalized MIM glyphs may be combined to form valid MIM diagrams. These rules are referred to as the syntax of the MIM notation; these are presented in Section 8.1. We then present additional syntactic rules such as those regarding the branching and the intermolecular glyphs. The primary source of information regarding the semantics (the meaning of each glyph and how it represents biological information) of each glyph is described in its definition; additional information about diagram semantics is presented in Section 8.4.

8.1 Interaction Syntax Rules

The syntax for MIM glyphs is presented in the tables of this section. MIM glyphs are required to follow the rules presented. Table 3 gives information on the way entities should be connected to interactions and Table 4 presents the rules for how interactions should be connected with other

interactions; refer to Section 4.2 for the definitions of "start", "on", and "end" for interaction glyphs.

Entries in the table below use the following notation for the interaction: a negative sign “-“ is used for invalid cases; a plus sign “+” is used for valid cases. There are three possible entries for “start”, “on”, and “end” for interaction glyphs separated by a “/”, if there are no valid cases then a single negative sign is used.

List of Acronyms:

- Entity acronyms: CE, conceptual entity; EC, explicit complex, EF, entity feature; IC, implicit complex; MO, modifier; RC, restricted copy; SP, simple physical entity; SS, source/sink.
- Interaction acronyms: AINH, absolute inhibition; CAT, catalysis; CIB, covalent irreversible binding; CLE, covalent bond cleavage; COMB, state combination; CVM, covalent modification; FFE, first feature connector; INH, inhibition; NCRB, non-covalent reversible binding; NFE, next feature connector; NSTI, necessary stimulation; PWOL, production without loss of reactants; STC, stoichiometric conversion; STI, stimulation; TMP, template.

Table 3: Entity-to-Interaction Connection Rules for MIM Entities and Interactions.

ENT/INT	NCRB	CIB	CVM	STC	PWOL/TMP	STI/NSTI/INH/AINH	CAT/CLE	FFE	NFE	COMB
EF	+/-/+	+/-/+	-/-/+	-	-	+/-/-	+/-/-	-/-/+	+/-/+	-
SP	+/-/+	+/-/+	-/-/+	+/-/+	+/-/+	+/-/-	+/-/-	+/-/-	-	-
IC	+/-/+	+/-/+	-/-/+	+/-/+	+/-/+	+/-/-	+/-/-	-	-	-
CE	-	-	-	-	-	+/-/+	-	-	-	-
MO	-	-	+/-/-	-	-	-	-	-	-	-
SS	-	-	-	+/-/+	+/-/+	-	-	-	-	-
RC	+/-/+	+/-/+	-	-	-	-	-	-	-	-
EC	+/+/+	+/+/+	-/+/-	+/-/+	+/-/-	+/-/-	+/-/-	-	-	+/+/+

Table 4: Interaction-to-Interaction Connection Rules for MIM Glyphs.

INT/INT	NCRB	CIB	CVM	STC	PWOL/TMP	STI/NSTI/INH/AINH	CAT/CLE	FFE	NFE	COMB
RB	-	-	-	-	-	-	-	-	-	-
CIB	-	-	-	-	-	-	-	-	-	-
CVM	-	-	-	-	-	-	-	-	-	-
STC	-	-	-	-	-	-	-	-	-	-
PWOL/TMP	-	-	-	-	-	-	-	-	-	-
STI/NSTI/ INH/AINH	-/-/+	-/-/+	-/-/+	-/-/+	-/-/+	-/-/+	-/-/+	-	-	-
CAT	-/-/+	-/-/+	-/-/+	-/-/+	-/-/+	-	-	-	-	-
CLE	-	-/-/+	-/-/+	-	-	-	-	-	-	-

8.2 Additional Syntactic Rules

The following syntactic rules also apply to formalized MIM diagrams.

- Interaction lines that are defined as have a line-terminating symbol only at one end of the line should not have a line-terminating symbol added to the other end of the line.

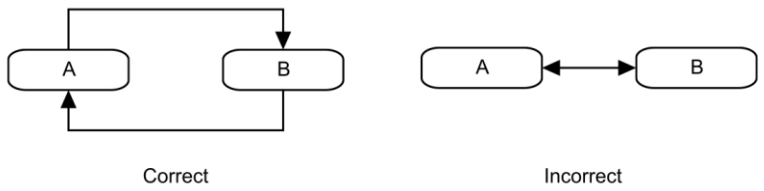

Figure 61: Proper method for reversing single arrowheaded interactions.

- A catalytic interaction or contingency carrying the intermolecular glyph must act on an interaction that has as an interactor that is common to both the catalytic interaction or contingency and the interaction being acted on; the common interactor may be a part of a complex.

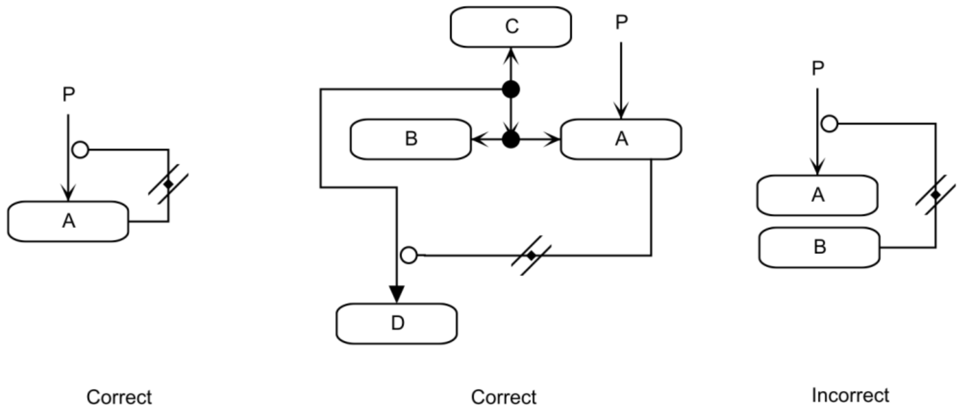

Figure 62: Examples of syntactically correct usage of the intermolecular glyph.

- Reactions with the intermolecular glyph can only involve a single entity.

- Reaction interaction types should not branch into any other interaction type.

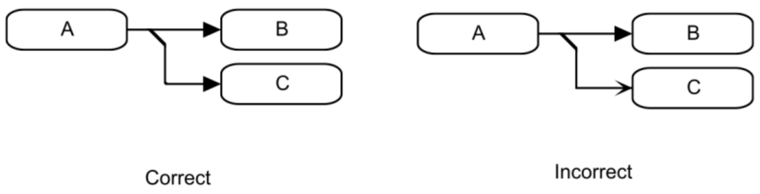

Figure 63: Examples of syntactically correct usage of reaction branching.

- Contingency and catalytic interaction types may branch into other non-reaction interaction types only if the resulting interaction is not double arrowheaded with another contingency or catalytic interaction, as shown Figure 64.

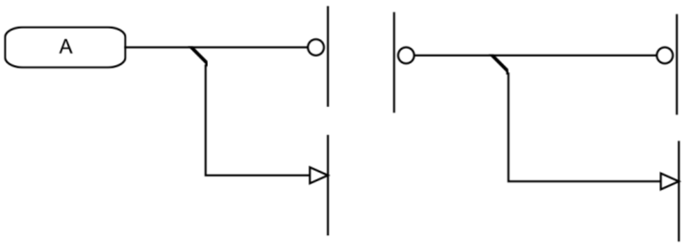

Figure 64: Branching of contingency and catalytic interactions.

- Simple physical entities should not possess multiple first feature glyphs, as shown in Figure 65.

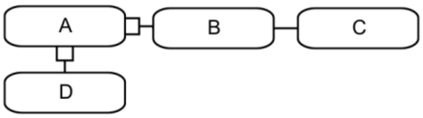

Figure 65: An invalid example showing a simple physical entity possessing multiple first entity features.

- The following interaction glyphs must not possess the intermolecular glyph: covalent modification, stoichiometric conversion, production without loss, template reaction, necessary stimulation, absolute inhibition, first feature connector, next feature connector, and state combination.

- Interactions originating from a conceptual entity glyph must not possess the intermolecular glyph.

- Several interaction types should not be branched, including: covalent modification, the first feature connector, and the next feature connector.

- The restricted copy entity cannot participate in any interaction type other than NCRB, and each restricted copy entity should only participate in a single reaction.

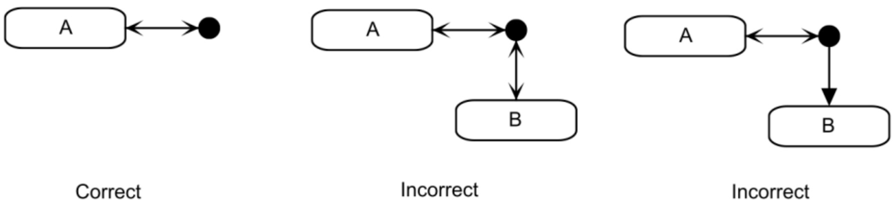

Figure 66: Examples of correct and incorrect restricted copy entity syntax.

8.3 Validation of MIM Diagrams

The rules described in Section 8 should be used to validate MIM diagrams.

8.4 Semantics in MIM

MIM diagrams can be viewed as a set of statements for the purpose of organizing biological knowledge. There is a limited capacity to infer additional statements from ones present on a diagram. The combined set of explicitly represented and inferred statements describes facts that make up a model of a biological system.

Models generated by the MIM notation do not describe the behavior of any specific instance of an entity, but rather present a set of rules that govern the behavior of instances if they exist. Entities in MIM imply pools of entities (see Section 6.1) where specific instances of a pool may participate in the various interactions of the given entity pool as outlined by the diagram. For instance, in Figure 1, in the pool of CaMK specific instances of CaMK may be catalyzing auto-phosphorylation reactions while others catalyze the phosphorylation of other various substrates. Whether these interactions occur is also dependent on the quantities and kinetics of all the entities needed for the interaction; the concepts of specific quantities and kinetics are outside the scope of this notation. The presence of a statement in a MIM diagram does not imply the following: 1) the presence of an observable in the natural world, 2) that an entity exists in sufficient quantities to carry out the activities described in a MIM diagram or 3) that an entity always exists in a state capable of behaving as indicated by a MIM diagram.

Truth-values are used in conjunction with the notation to build relationships between propositions. Statements regarding entities are generated through glyphs of the notation that are explicitly represented on a diagram and carry a truth-value of "TRUE". Entities that are inferred (see Section 8.4.1) carry a truth-value of "INFERRED" which is equivalent to "TRUE" and is differentiated only for the purpose of illustrating which entities explicitly exist on a diagram and from those that are inferred. Statements that exist on a diagram or have been inferred must resolve to truth-values that are either "TRUE" or "FALSE" within the model. Statements that are not represented on the diagram and cannot be inferred carry a truth-value of "UNKNOWN" in the model. This view of the model represents an open-world view of the model described by the diagram. For instance, under the open-world assumption, if a diagram states the single fact that entity A binds to B, then one can only say that it is unknown whether entity C binds to A or B, but the possibility cannot be ruled out.

One of the simplest statements in MIM can be generated by diagramming a simple physical entity (SPE). For instance, diagramming an SPE labeled A indicates a truth-value for the entity

A both as a statement and in the model, as shown in Table 5 where statement column refers to a graphical component on the diagram or an inferred component, and the model column refers to any alterations in the semantics of presented statements given other interactions.

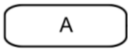

Figure 67: A simple physical entity labeled "A".

Table 5: Truth-value for a diagram containing only a SPE labeled "A".

Entity	Statement	Model
**A**	TRUE	TRUE

Binding interactions are statements that generate entities with "TRUE" truth-values.

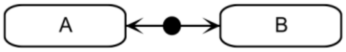

Figure 68: A complex resulting from the binding between simple physical entities A and B.

Table 6: Truth-values for a diagram containing a non-covalent reversible binding of entities A and B.

Entity	Statement	Model
**A**	TRUE	TRUE
**B**	TRUE	TRUE
**A:B**	TRUE	TRUE

Binding interactions do not possess a transitive binding property in that binding interactions do not affect other binding interactions indirectly (see Section 10.1).

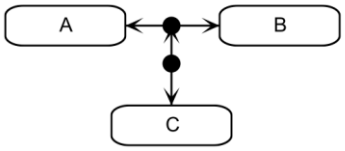

Figure 69: A trimer between simple physical entities A, B, and C.

Table 7: Truth-values for a diagram containing only a trimer between simple physical entities A, B, and C.

Entity	Statement	Model
**A**	TRUE	TRUE
**B**	TRUE	TRUE
**C**	TRUE	TRUE
**A:B**	TRUE	TRUE

**A:C**	FALSE	UNKNOWN
**B:C**	FALSE	UNKNOWN
**A:B:C**	TRUE	TRUE

The truth-value of entities or interactions that are not explicitly presented on a MIM diagram or inferred from the diagram is "UNKNOWN".

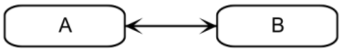

Figure 70: Example to illustrate truth-values for entities not appearing in a diagram in Table 8.

Table 8: Truth-values for entities in Figure 70.

Entity	Statement	Model
**A:B**	TRUE	TRUE
**A:C**	FALSE	UNKNOWN
**X**	FALSE	UNKNOWN
**X:Y**	FALSE	UNKNOWN

It is a goal of the MIM notation that all readers of MIM diagrams should be able to enumerate all statements appearing on a diagram. And from these statements, readers should be able to derive which facts in the model either are known to exist, are known to not exist, or whether their existence is unknown.

8.4.1 Inference Cases in MIM

There are several cases in which MIM entities are inferred that are not directly represented on a MIM diagram. There are two MIM glyphs that may result in inferred MIM entities: necessary stimulation (Section 7.3.2) and absolute inhibition (Section 7.3.4) when targeting binding interactions. Note: this section makes use of the acronyms defined in Section 8.1.

Basic Inference Cases

Statements regarding entities that are the products of interactions are subject to contingencies that target the interactions. In a simple inference case involving two binding interactions where the binding interactions are connected by a single necessary stimulation (as shown in Figure 71) or absolute inhibition (as shown in Figure 72) a change is made to the semantics of the targeted interaction.

A necessary stimulation causes the truth-value to be "TRUE" in the model for an entity possessing the three components presented by the two binding interactions even though it is not directly represented in the diagram. Such an inference is marked as "INFERRED" in the "Statement" column of Table 9. A second change is made to the semantics in that even though there is a statement explicitly representing a complex of A and B, the case results as "FALSE" in the model since such a complex is only permitted if entity A is first phosphorylated. In the right diagram of Figure 71, an illustration is given of the interaction that would need to exist for the

entity A.P:B to be explicitly stated and would produce the entity that is inferred by the necessary stimulation.

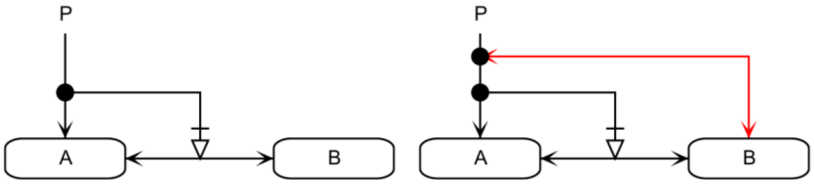

Figure 71: Inference case using necessary stimulation. The red interaction on the right indicates the interaction that would directly state the entity inferred through the necessary stimulation.

Table 9: Truth-values for entities in the left diagram of Figure 71.

Entity	Statement	Model
**A.P**	TRUE	TRUE
**A:B**	TRUE	FALSE
**A.P:B**	INFERRED	TRUE

An absolute inhibition causes the truth value to be "FALSE" in the model for an entity possessing the three components presented by the two binding interactions even though it is not directly represented in the diagram. This inference is also marked as "INFERRED" in the "Statement" column of Table 10 since the interaction explicitly representing the entity is not present; this interaction that is prevented is shown in the right diagram of Figure 72.

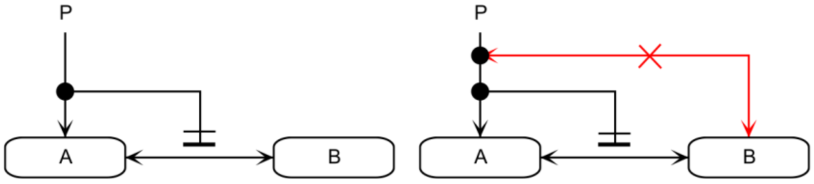

Figure 72: Inference case using absolute inhibition. The red interaction on the right indicates the interaction that is being inferred to being prevented through the absolute inhibition.

Table 10: Truth-values for entities in the left diagram of Figure 72.

Entity	Statement	Model
**A.P**	TRUE	TRUE
**A:B**	TRUE	TRUE
**A.P:B**	INFERRED	FALSE

A key difference between the semantics of absolute inhibition and necessary stimulation is the change in the semantics of the targeted interaction, as illustrated in Table 9 and Table 10 for the

complex, A:B, between the unphosphorylated entity A and B; the complex A:B has a truth-value "TRUE" for the case of absolute inhibition, but "FALSE" for the case necessary stimulation.

Propagation of Semantics

These changes in the semantics of an interaction are propagated to other interactions that may be the source or target of the interaction with altered semantics. In the case shown in Figure 73, the explicit complex labeled "x" is the complex of phosphorylated A bound to B and C. The complex "x" does not represent the complex of unphosphorylated A bound to B and C; this is also illustrated in Table 11.

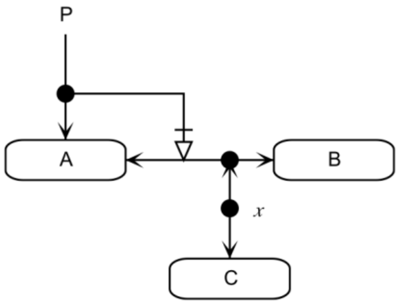

Figure 73: Semantic propagation. The entity labeled "x" represents a complex of phosphorylated A bound to B and C.

Table 11: Truth-values for entities in Figure 73.

Entity	Statement	Model
**A.P**	TRUE	TRUE
**A:C**	FALSE	UNKNOWN
**B:C**	FALSE	UNKNOWN
**A.P:B**	INFERRED	TRUE
**A.P:B:C**	INFERRED	TRUE
**A:B:C**	TRUE	FALSE

There may also be more advanced inference cases where a necessary stimulation or an absolute inhibition do not directly target an immediate binding interaction possessing the entity as a direct source, as shown in Figure 74, but the entity being addressed by the inference forms part of the complex acting as an interactor in the targeted binding interaction. In such a case as shown in Figure 74, only the interaction being targeted possesses altered semantics. This is illustrated in Table 12, in that the complex of A:B:C, the entity A must be phosphorylated, yet the semantics of the complex A:B remained unchanged in that there is no requirement made on entity A to be phosphorylated.

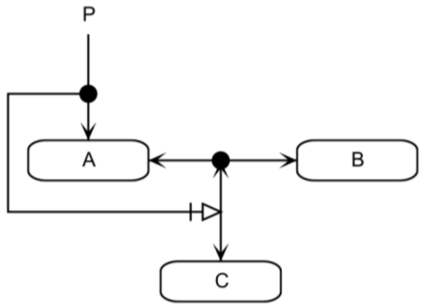

Figure 74: Inference case when an interaction does not directly target an immediate binding interaction.

Table 12: Truth-values for entities in Figure 74.

Entity	Statement	Model
**A.P:B:C**	INFERRED	TRUE
**A:B:C**	TRUE	FALSE
**A:B**	TRUE	TRUE

Multiple Contingency Inferences

More advanced inference cases may arise when multiple contingencies target a single interaction. In such cases, all the inferences must be resolved. This resolution is independent of the order in which the contingencies are chosen. Entities with a truth-value of "TRUE" will be those that resolve all the inference cases. Even though an entity may possess a truth-value of "FALSE" in the model, it may still act as the source of inferences (see Figure 77). Inference cases involving multiple contingencies may not always lead to semantically valid conclusions (see Section 8.4.2). There still maybe justification for depicting such cases, for example, they may serve the purpose; illustrating a controversy existing in scientific literature related to the system, for example.

Figure 75 illustrates multiple inferences made regarding a single interaction. The interaction emanating from A.P1 causes the truth-value of A.P1:B in the model to be "FALSE" while A.P2 causes the truth-value of A.P2:B in the model to be "FALSE". Resolved together, these interactions causes the truth-value of A.P1.P2:B to be "FALSE" in the model, as illustrated in Table 13.

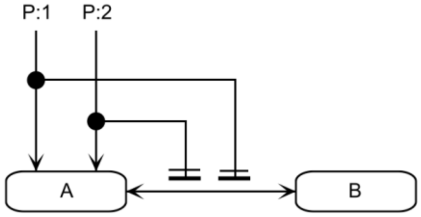

Figure 75: Inference case when multiple inferences are made regarding a single interaction.

Table 13: Truth-values for entities in Figure 75.

Entity	Statement	Model
**A.P1**	TRUE	TRUE
**A.P2**	TRUE	TRUE
**A:B**	TRUE	TRUE
**A.P1:B**	INFERRED	FALSE
**A.P2:B**	INFERRED	FALSE
**A.P1.P2:B**	INFERRED	FALSE

Another case that may arise is where two contingencies target interactions where the product of the interaction is the source the contingency; Figure 76 and Figure 77 illustrate the case when contingencies are of the same type, while Figure 78 illustrates the case where they are different.

The case with two absolute inhibitions is shown in Figure 76. As shown in Table 14, the inferred complex A:B:C is prevented while complexes A:B and A:C follow from semantics detailed for the basic cases.

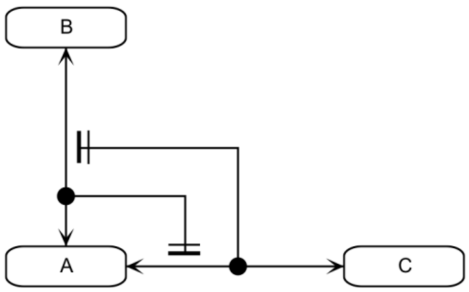

Figure 76: Mutual absolute inhibition inference case.

Table 14: Truth-values for entities in Figure 76.

Entity	Statement	Model
**A:B**	TRUE	TRUE
**A:C**	TRUE	TRUE
**A:B:C**	INFERRED	FALSE

The case with two necessary stimulations is shown Figure 77. Taken independently the necessary stimulation labeled "x" causes the truth-value of complex A:B to be "FALSE" in the model, while the necessary stimulation labeled "y" causes the truth-value of complex A:C to be "FALSE" in the model; these are the same cases as in Figure 71. Each inference resulting from a necessary stimulation also results in the entity A:B:C. Since each inference carries a different ordering mechanism for how the complex is formed, both ordering mechanisms should be assumed; for example, NSTI labeled "x" indicates that entity A must first bind to entity C prior to binding to B. When these two contingencies are resolved together only the case of A:B:C satisfies both independent inferences causing the truth-value to be "TRUE" in the model.

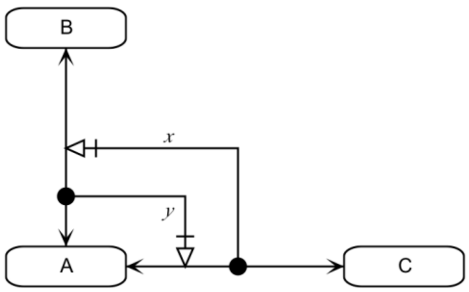

Figure 77: Mutual necessary stimulation inference case.

Table 15: Truth-values for entities in Figure 77.

Entity	Statement	Model
**A:B (for NSTI "x")**	TRUE	FALSE
**A:C (for NSTI "y")**	TRUE	FALSE
**A:B:C**	INFERRED	TRUE

Figure 78 illustrates multiple contingencies that result in a semantic invalidity. For the case of complex A:B, the necessary stimulation and absolute inhibition result in contradictory truth-values within the model shown in Table 16. The source of the absolute inhibition is the complex A:B. The complex A:B carries a truth-value "FALSE" from the necessary stimulation. If the complex A:B is prevented from occurring by the necessary stimulation, then there can be no possibility for it to be the source of the absolute inhibition. The source of the absolute inhibition is not the complex A:B:C, since this is not indicated on the diagram. Since this diagram is semantically invalid the truth-value for inferred complex A:B:C is not shown.

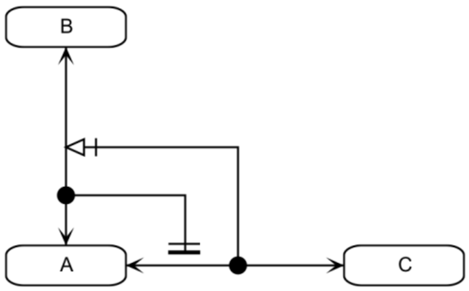

Figure 78: Opposing mutual inferences case.

Table 16: Truth-values for entities in Figure 78.

Entity	Statement	Model
**A:B (for NSTI)**	TRUE	FALSE
**A:C (for NSTI)**	TRUE	TRUE
**A:B (for AINH)**	TRUE	TRUE
**A:C (for AINH)**	TRUE	TRUE

Figure 79 shows two diagrams with cases where a single common entity is not shared either through direct binding interactions or by a complex. In this case there is no alteration of the semantics of the targeted interaction as shown in Table 17. In either the left or right diagram of Figure 79, the truth-values of the entities should be interpreted as if the absolute inhibition or necessary stimulation were not present.

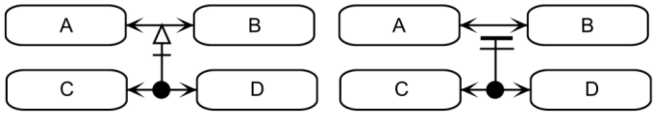

Figure 79: Inference cases where there is no common entity between the source and the targeted interactions.

Table 17: Truth-values for entities in Figure 79.

Entity	Statement	Model
**A:B (for NSTI diagram)**	TRUE	TRUE
**C:D (for NSTI diagram)**	TRUE	TRUE
**A:B (for AINH diagram)**	TRUE	TRUE
**C:D (for AINH diagram)**	TRUE	TRUE

8.4.2 Semantic Validity of MIM Diagrams

Contradictory statements can result from inferred statements within a diagram. MIM diagrams may be considered semantically invalid if there are contradictory statements within a diagram or if there are inconclusive statements. Figure 80 shows a case with a contradictory statement arising from the usage of both an absolute inhibition and necessary stimulation. Table 18 is a merging of Table 9 and Table 10; the tables for the individual (i.e. necessary stimulation and absolute inhibition) interactions.

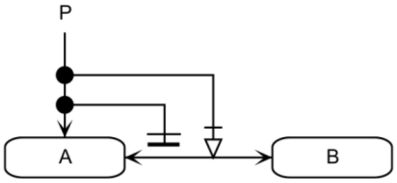

Figure 80: Contradictory inference example.

Table 18: Truth-values for entities in Figure 80.

Entity	Statement	Model
**A.P:B (for NSTI)**	INFERRED	TRUE
**A.P:B (for AINH)**	INFERRED	FALSE
**A:B (for AINH)**	TRUE	TRUE

**A:B (for NSTI)**	TRUE	FALSE

Cases where an absolute inhibition targets a binding interaction in which the source entity of the absolute inhibition is also an interactor or product of the targeted interaction are semantically invalid; the contradiction is shown in Table 19.

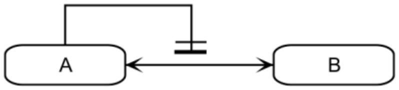

Figure 81: Inference cases where an absolute inhibition targets a binding interaction in which the source entity of the absolute inhibition is also the source or product of the targeted interaction.

Table 19: Truth-values for entities in Figure 81.

Entity	Statement	Model
**A:B (from NCRB)**	TRUE	TRUE
**A:B (from AINH)**	INFERRED	FALSE

8.4.3 Relation to Previously Described Interpretations of MIM Diagrams

The interpretation of MIM diagrams outlined in this section most closely conforms to the heuristic interpretation of the MIM notation rather than the explicit or combinatorial interpretations that were previously outlined (Kohn, Aladjem et al. 2006). The truth-values in the “Statement” column of the MIM diagram examples, however, most closely correspond to the previously described explicit interpretation of the MIM notation. The explicit interpretation carries not only specific semantics, but also restrictions on the types of glyphs that a diagram may use; this limited subset of glyphs simplifies the conversion of MIM diagrams to computational models. Readers intending to use MIM diagrams for computational simulations should refer to the previous publication (Kohn, Aladjem et al. 2006). The set of entities with truth-values "TRUE" (or "INFERRED" since it is equivalent to "TRUE") should be used wherever the previously described explicit interpretation is required for use cases such as computational modeling.

9. Layout Guidelines of MIM Diagrams

The components of a MIM diagram may be placed on a diagram in a manner to emphasize messages authors wish to convey. There is no pre-determined arrangement for the components (considered a layout) of a MIM diagram and various layouts may be applicable to a given set of entities and interactions. This section provides layout guidelines for MIM diagrams: requirements are rules that should be followed and recommendations are rules that may be followed. Several of these rules were first described in the SBGN Entity Relationship specification (Le Novère 2009). These guidelines apply to all MIM diagrams regardless of the method used to create them.

9.1 Layout Requirements

Valid MIM diagrams must follow all layout requirements.

9.1.1 Entity-entity overlap

Entity glyphs should not overlap as shown in Figure 82.

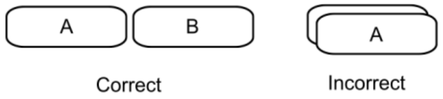

Figure 82: Non-overlapping entities.

9.1.2 Arrowhead overlap

Glyphs should not overlap the arrowheads of an interaction line as shown in Figure 83.

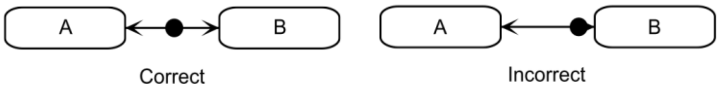

Figure 83: Dot glyph overlap of interaction arrowhead.

9.1.3 Interaction-interaction overlap

Interactions should not overlap other interactions.

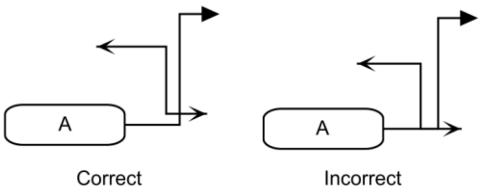

Figure 84: Overlap of two interactions

9.1.4 Color usage

Glyph semantics are not affected by color.

9.2 Layout Recommendations

Recommendations are layout guidelines that have been developed over the history of the MIM notation that should be followed unless there is a compelling reason to do otherwise.

9.2.1 Entity labels

It is recommended that all entities possess labels; the lack of labels may create ambiguity for readers. It is also recommended that entity labels used standardized nomenclatures when possible, such as HUGO names for human genes (http://www.genenames.org).

9.2.2 Interaction annotation labels

It is recommended that all interactions possess annotation labels (see Section 7.1.2) that reference the specific sources that were used to obtain the interaction information.

9.2.3 Label orientation

The labels of entities are recommended to be oriented horizontally or vertically.

9.2.4 Single line labels

The labels of entities are recommended to appear on a single line. This recommendation is made to simplify the implementation of MIM in software editors.

9.2.5 Interaction line routing

MIM diagrams have historically followed an orthogonal layout convention. In this convention, changes in direction of an interaction glyph should occur at 90°. Lines should not change direction by more than 90° to avoid ambiguity with branch points. Interaction lines may cross when necessary; it is recommended that lines cross at 90°.

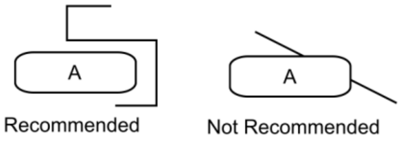

Figure 85: Interaction line routing

9.2.6 Node duplication

It is recommended that enclosed and labeled entity glyphs appear once per diagram to maintain the idea of traceability whereby all the interactions of an entity are routed to a single point on a diagram. This recommendation facilitates the creation of traceable diagrams in which interactions can be traced back to a single point on the diagram and helps to reduce diagram complexity. Other entities, such as modifiers and source/sink entities, do not carry this recommendation because they may have high prevalence in MIM diagrams that would lead to an overly connected and potentially confusing diagram.

9.2.7 Ordering of entity features

It is recommended that entity features be placed in a numerically sequential order with increasing numerical values of the labels. Additionally, it is recommended that entity features be labeled using the conventions of the entities they represent (e.g. N-to-C terminus for proteins). For entity features for polymers of nucleic acids it is recommended that they be represented from the 5’ to 3’ end. These recommendations are directly related to the semantics of the first and next features semantics (see Section 7.5).

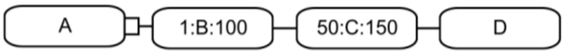

Figure 86: Recommended method for laying out entity features. Entity features B and C overlap in position, and semantically the assertion is made that entity feature D exists after entity feature C though its location is not represented.

9.2.8 Labeling of covalent modifications

It is recommended that labels for covalent modifications use one of the following conventions: 1) a single integer value or 2) a single amino acid prefix followed by an integer. Other formats may not be well described enough for interpretation or translation.

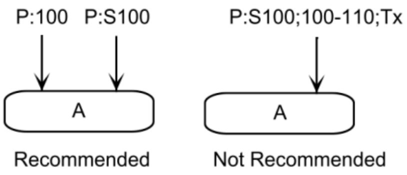

Figure 87: Recommended formats for position labels of covalent modifications

9.2.9 Label overlap

It is recommended that labels do not overlap other labels.

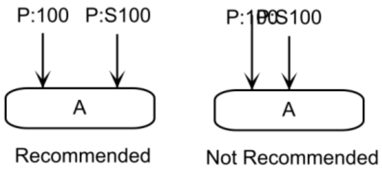

Figure 88: Covalent modification position label overlap.

9.2.10 Minimizing line length

It is recommended that entities sharing interactions be placed near each other.

10. Known Issues

10.1 Interpretation Modes

Kohn and co-authors describe an additional interpretation mode for the MIM notation that alter the semantics of entities (Kohn, Aladjem et al. 2006). The "combinatorial" mode assumes that possible interactions do in fact occur by making use of transitivity for interactions. This interpretation mode remains to be formally developed as a general extension of the previous description.

10.2 Glyph Set

10.2.1 Expanded Concepts

This level of the specification utilizes core glyphs of the 2006 publication for the MIM notation (Kohn, Aladjem et al. 2006). This specification does not include glyphs and their related concepts from publications post-2006. Polymerization and helicase interaction glyphs were introduced in a recent 2009 publication (Kohn, Aladjem et al. 2009). As the syntax and semantics of these glyphs is clarified, the glyphs will be added to later levels of this specification. This specification also does not support several features of interest including:

compartmentalization of entities, the transport of entities, and representation of stoichiometric coefficients.

10.2.2 Shorthand Glyphs

The current specification restricts usage of some visual shortcuts outline in Kohn 2006. Some of these shortcuts may be added to later versions of the specification. For instance the 2006 MIM specification includes the use of double-headed contingency interactions to describe cooperative and mutually exclusive binding in Figure 4 of the 2006 publication by Kohn and co-authors (Kohn, Aladjem et al. 2006). A double arrowheaded contingency glyph should be replaced by two single-arrowheaded contingencies glyphs from this specification.

11. Quick Reference Sheet

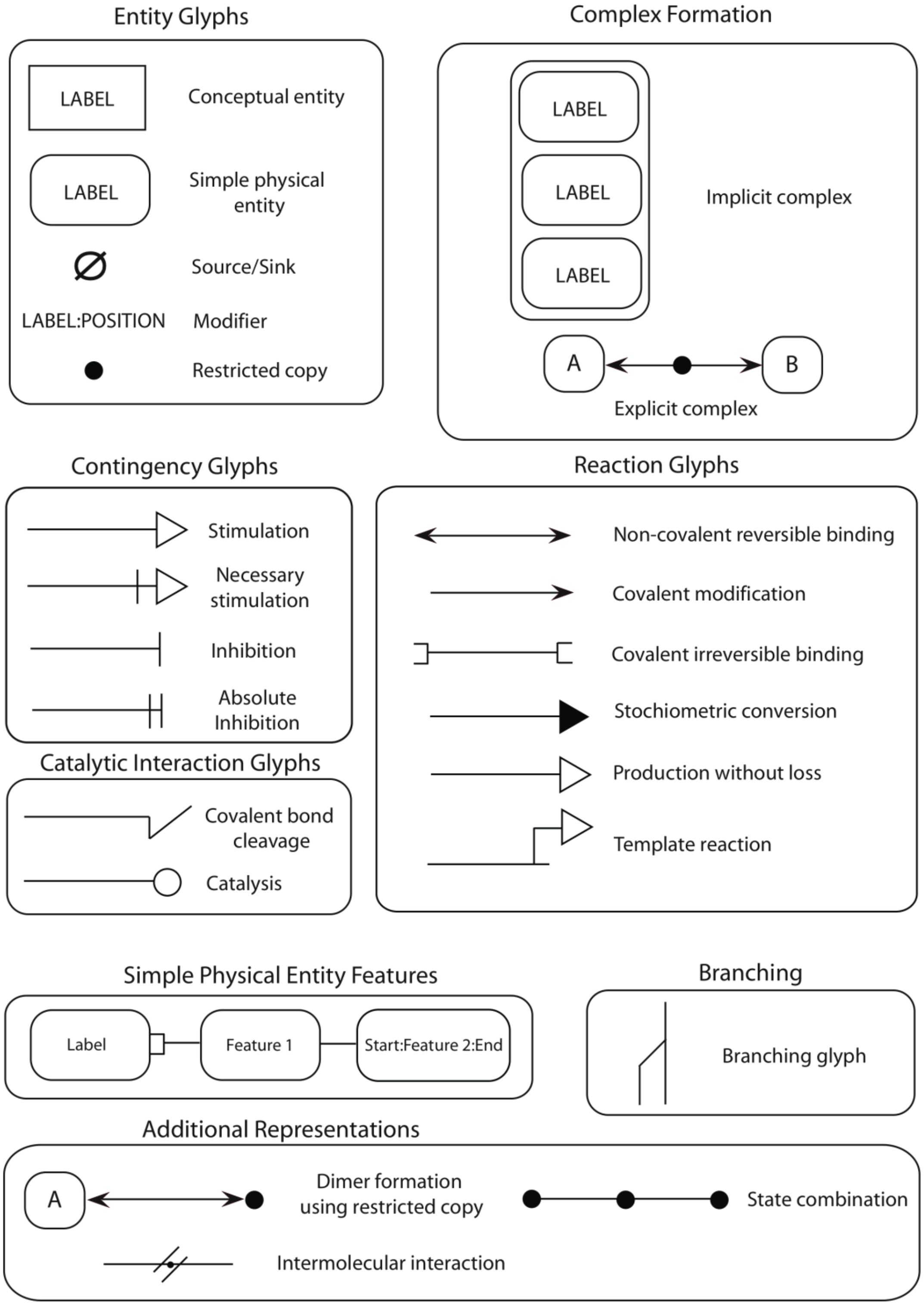
Entity Glyphs
LABEL
Conceptual entity
LABEL
Simple physical entity
Source/Sink
LABEL:POSITION
Modifier
Restricted copy
Complex Formation
LABEL
LABEL
LABEL
Implicit complex
A
B
Explicit complex
Contingency Glyphs
Stimulation
Necessary stimulation
Inhibition
Absolute Inhibition
Catalytic Interaction Glyphs
Covalent bond cleavage
Catalysis
Reaction Glyphs
Non-covalent reversible binding
Covalent modification
Covalent irreversible binding
Stochiometric conversion
Production without loss
Template reaction
Simple Physical Entity Features
Label
Feature 1
Start:Feature 2:End
Branching
Branching glyph
Additional Representations
A
Dimer formation using restricted copy
State combination
Intermolecular interaction

12. Revision History

- Level 1 - Initial specification

13. Acknowledgements

13.1 Main Contributors

Augustin Luna, Evrim I. Karac, Margot Sunshine, Lucas Chang, Mirit I. Aladjem and Kurt W. Kohn

13.2 Acknowledgements

We would like to acknowledge discussions with Can Ozturan, Mine Edes, Turkan Haliloglu, Ruth Nussinov and Martijn van Iersel. Additionally, discussions with members of the SBGN and BioPAX communities were useful to the success of this project, especially Emek Demir, Nicolas Le Novère, and Hiroaki Kitano.

14. References

Aladjem, M. I., S. Pasa, et al. (2004). "Molecular interaction maps--a diagrammatic graphical language for bioregulatory networks." Sci. STKE **2004**: pe8.

Bradner, S. (1997). Key words for use in RFCs to Indicate Requirement Levels, IETF.

Demir, E., M. P. Cary, et al. (2010). "The BioPAX community standard for pathway data sharing." Nature biotechnology **28**: 935-42.

Kohn, K. W. (1999). "Molecular interaction map of the mammalian cell cycle control and DNA repair systems." Mol. Biol. Cell **10**: 2703-2734.

Kohn, K. W. (2001). "Molecular interaction maps as information organizers and simulation guides." Chaos **11**: 84-97.

Kohn, K. W., M. I. Aladjem, et al. (2006). "Depicting combinatorial complexity with the molecular interaction map notation." Molecular systems biology **2**: 51.

Kohn, K. W., M. I. Aladjem, et al. (2006). "Molecular interaction maps of bioregulatory networks: a general rubric for systems biology." Mol. Biol. Cell **17**: 1-13.

Kohn, K. W., M. I. Aladjem, et al. (2009). "Network architecture of signaling from uncoupled helicase-polymerase to cell cycle checkpoints and trans-lesion DNA synthesis." Cell Cycle **8**: 2281-2299.

Le Novere, N. (2006). "Model storage, exchange and integration." BMC Neurosci **7 Suppl 1**: S11.

Le Novère, N. (2006). "Model storage, exchange and integration." BMC neuroscience **7 Suppl 1**: S11.

Le Novère, N., Moodie, Stuart, Sorokin, Anatoly, Schreiber, Falk, and Mi, Huaiyu (2009). Systems Biology Graphical Notation: Entity Relationship language Level 1.

Moodie, S., Le Novere, Nicolas , Sorokin, Anatoly , Mi, Huaiyu, and Schreiber, Falk (2009). Systems Biology Graphical Notation: Process Description language Level 1.
